# SPP1‐driven spatial niche remodelling defines aggressive progression in the basal subtype of upper tract urothelial carcinoma

**DOI:** 10.1002/ctm2.70782

**Published:** 2026-08-24

**Authors:** Qi Zhang, Zhipeng Wen, Ziyang Yang, Xuezhi Long, Jieshen Xian, Yueting Huang, Yufei Liu, Yubo Wang, Jiahao Cheng, Gengjia Chen, Xuekun Zhang, Tuxiong Huang, Di Gu

**Affiliations:** ^1^ Department of Urology The First Affiliated Hospital Guangzhou Medical University Guangzhou China; ^2^ Guangdong Provincial Key Laboratory of Urological Diseases Guangzhou Medical University Guangzhou China; ^3^ Nanshan School Guangzhou Medical University Guangzhou China

**Keywords:** basal subtype, myofibroblastic cancer‐associated fibroblasts, SPP1, single‐cell RNA sequencing, spatial heterogeneity, tumour microenvironment, tumour‐associated macrophages, tumour angiogenesis, upper tract urothelial carcinoma

## Abstract

**Background:**

Upper tract urothelial carcinoma (UTUC) is a rare, aggressive malignancy representing 5%‒10% of urothelial carcinomas. Its basal subtype remains understudied but is preferentially associated with muscle invasion and poorer disease‐specific survival (DSS). Although the genomic landscape of UTUC has been extensively profiled, current bulk transcriptomic classification cannot resolve cellular heterogeneity or spatial organisation, potentially obscuring subtype‐specific microenvironmental programs.

**Methods:**

We integrated single‐cell RNA sequencing and spatial transcriptomics to resolve relationships among molecular subtype, muscle invasion and cellular heterogeneity within the UTUC microenvironment. Cellular communication and multiscale spatial analyses identified spatial programs, with tissue‐level validation by multiplex immunofluorescence. These programs were further examined in external bladder cancer datasets spanning single‐cell, spatial and GeoMx platforms. SPP1 knockdown in J82 cells tested epithelial‐intrinsic effects. We evaluated the subtype‐discriminatory and prognostic relevance of SPP1 in the Japan‐UTUC cohort.

**Results:**

Basal UTUC exhibited an immune‒stromal‐enriched microenvironment primarily associated with molecular subtype rather than pathological stage. Within basal tumours, muscle invasion coincided with increased heterogeneity and two spatial programs. A conserved SPP1^+^ tumour‐associated macrophage‒FAP^+^ myofibroblast boundary program was associated with myeloid immunosuppression and extracellular matrix remodelling. External datasets provided convergent support for its components and localisation near basal‐high epithelium at invasive margins. A more heterogeneous angiogenic program involved VEGFA^+^ tumour‐associated neutrophil‒CXCR4^+^ tip endothelial interactions. At the epithelial level, SPP1 knockdown reduced J82 cell viability, migration and invasion. SPP1 discriminated basal from luminal tumours (area under the receiver operating characteristic curve (AUC) = .839), and higher expression remained associated with poorer DSS after pathological stage adjustment (hazard ratio = 1.17, 95% confidence interval = 1.01‒1.35, *p* = .036).

**Conclusions:**

Basal UTUC has a largely stage‐independent, immune‒stromal‐enriched background that is spatially reorganised with muscle invasion, with an SPP1‐associated myeloid‒stromal boundary program as its most reproducible feature. Spatial, functional and clinical evidence supports the prognostic value of SPP1 and warrants its evaluation as a therapeutic target.

## Key points

- Basal UTUC has a largely stage‐independent immune–stromal background that is spatially reorganised with muscle invasion.
- The SPP1^+^ TAM–FAP^+^ myofibroblastic CAF (myCAF) program is enriched at invasive boundaries and associated with ECM remodelling and myeloid suppression, whereas the angiogenic program is more heterogeneous.
- Spatial, functional and clinical evidence supports SPP1 as a prognostic biomarker and candidate therapeutic target.

## Graphical abstract

Basal upper tract urothelial carcinoma has a largely stage‐independent immunestromal background that is spatially reorganized with muscle invasion into an SPP1‐associated myeloidstromal boundary program and a more heterogeneous angiogenic program. Spatial, functional, and clinical evidence supports SPP1 as a prognostic biomarker and candidate therapeutic target.

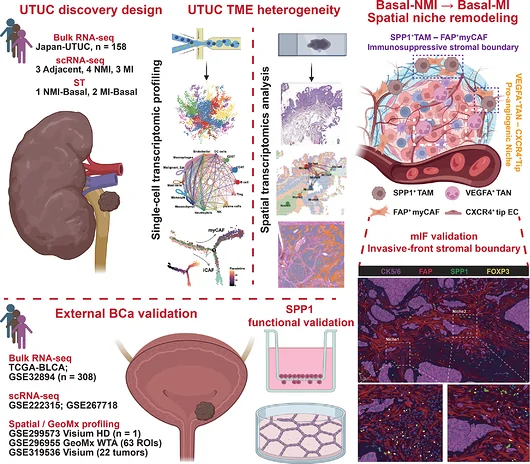

## 1 INTRODUCTION

Upper tract urothelial carcinoma (UTUC) arises from the urothelium of the renal pelvis or ureter and represents a rare subtype of urothelial carcinoma (UC), accounting for approximately 5%‒10% of all UC.^1^ Compared with bladder cancer (BCa), UTUC often presents with a more aggressive clinical phenotype, with nearly 60% of patients presenting with muscle‐invasive (MI) tumours at initial diagnosis, a pathological status associated with poor prognosis.^1^, ^2^

Although the genomic landscape of UTUC has been largely characterised, genomic alterations alone explain only part of its pathogenesis.^3^, ^4^, ^5^ Recent genomic profiling of 122 Chinese patients with UTUC identified recurrent KMT2D, TERT promoter, FGFR3 and TP53 alterations, together with genomic differences across populations and between renal pelvis and ureteral tumours.^6^ Relative to BCa, UTUC typically exhibits a lower tumour mutational burden and fewer TP53 mutations, whereas FGFR alterations are enriched and support biomarker‐guided FGFR‐targeted therapy in advanced disease.^3^, ^5^, ^7^ By contrast, transcriptomic features may better reflect tumour‒immune functional states and show higher concordance between primary and metastatic UTUC lesions, highlighting the importance of resolving tumour microenvironment (TME) heterogeneity.^8^

Current UTUC subtyping largely adopts the BCa classification framework, with luminal‐related subtypes predominating and accounting for more than 80% of cases across different cohorts, typically characterised by low T‐cell infiltration and weak immune activity, consistent with an immune‐desert phenotype and a relatively favourable prognosis.^8^, ^9^, ^10^ In contrast, basal UTUC constitutes a smaller proportion overall but is enriched among MI tumours and exhibits greater aggressiveness, thereby contributing disproportionately to UTUC‐related mortality.^9^, ^11^

However, as noted by Kim et al., current UTUC subtyping studies largely rely on bulk transcriptomics, whose limited resolution hampers deconvolution of tumour, stromal and immune cell contributions and may confound subtype signals.^11^ Moreover, the extent to which molecular subtype and pathological stage shape the composition and spatial organisation of the UTUC TME remains unclear. This knowledge gap is particularly evident for basal UTUC, which has received far less attention than luminal disease and remains insufficiently characterised, with its cellular heterogeneity, spatial architecture and potential therapeutic vulnerabilities still poorly defined.

To address these questions, we integrated internal single‐cell RNA sequencing (scRNA‐seq) and spatial transcriptomic (ST) data with the external Japan‐UTUC bulk cohort (*n* = 158).^3^ These analyses showed that the immune‒stromal‐enriched background of basal UTUC was primarily associated with molecular subtype rather than pathological stage. Within basal tumours, muscle invasion was associated with spatial reorganisation of this background into an SPP1‐associated macrophage‒fibroblast boundary program and a more heterogeneous neutrophil‒endothelial angiogenic program. At invasive margins, the coexistence of basal‐high epithelium with an SPP1‐associated program characterised by myeloid suppression and extracellular matrix (ECM) deposition was conserved across external BCa datasets. Separately, J82 loss‐of‐function assays supported an epithelial‐intrinsic role for SPP1 in malignant behaviour. SPP1 further distinguished the basal phenotype comparably to GATA3 and CK5/6 and refined prognostic stratification beyond pathological stage, supporting its potential as a prognostic biomarker and therapeutic target.^12^

## 2 RESULTS

### 2.1 Basal UTUC exhibits a stage‐independent immune‒stromal‐enriched landscape

**FIGURE 1:**
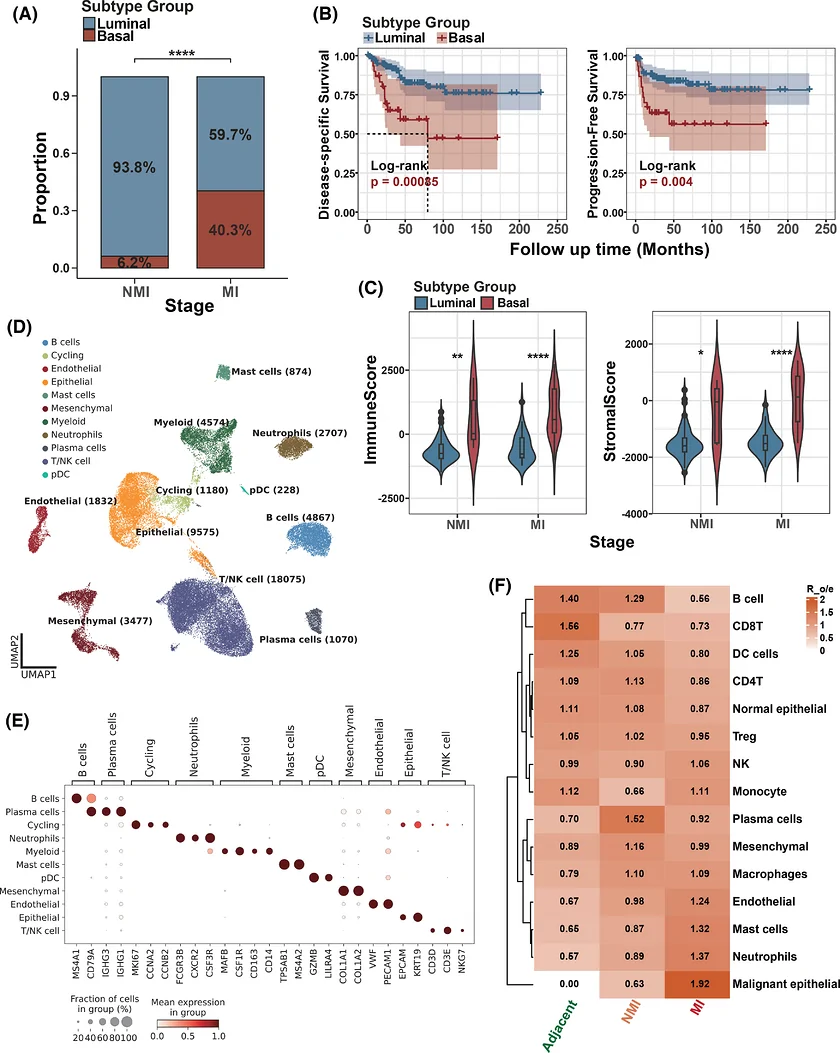
Molecular subtype, pathological stage and tumour microenvironment in upper tract urothelial carcinoma (UTUC). (A) Proportions of luminal and basal tumours among non‐muscle‐invasive (NMI) and muscle‐invasive (MI) cases in the Japan‐UTUC cohort. (B) Kaplan‒Meier curves for disease‐specific survival (DSS; left) and progression‐free survival (PFS; right), stratified by molecular subtype. (C) ImmuneScore (left) and StromalScore (right) by molecular subtype within the NMI and MI groups. (D) uniform manifold approximation and projection (UMAP) of the internal single‐cell RNA sequencing cohort showing the major annotated cell compartments. (E) Representative markers used for cell‐type annotation; dot size indicates the fraction of cells expressing each gene, and colour indicates mean expression. (F) Ratio of observed to expected (*R*_o/e_) cell abundance in adjacent tissues, NMI tumours and MI tumours. Statistical annotations are shown in the corresponding panels.

In the Japan‐UTUC cohort, luminal tumours predominated, whereas the basal subtype was enriched in MI cases and associated with worse survival (Figures 1A,B and S1A). When stratified by stage, basal tumours showed higher ImmuneScore and StromalScore than luminal tumours in both non‐muscle‐invasive (NMI) and MI groups, together with upregulation of immune activation, immune suppression, myeloid and stromal gene signatures. Interaction analysis indicated that this immune‒stromal‐enriched phenotype was primarily linked to molecular subtype rather than stage (Figures 1C and S1B,C and Table S1).

**FIGURE 2:**
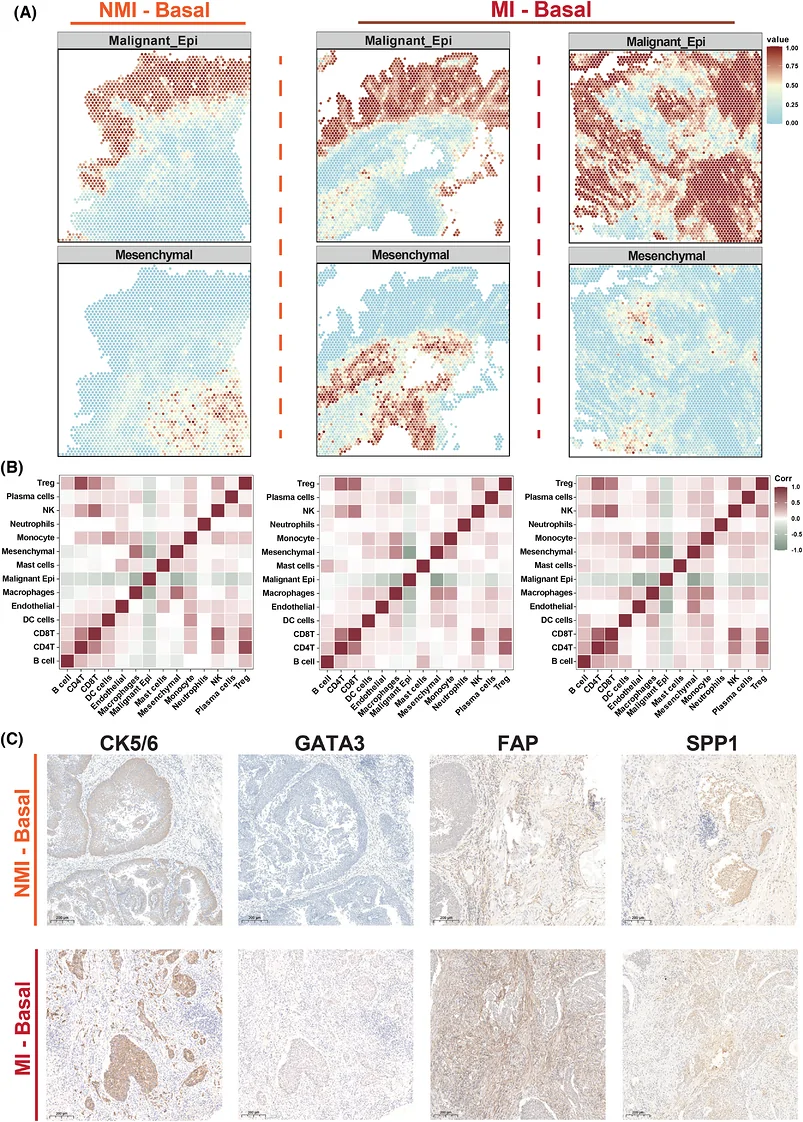
Spatial transcriptomic analysis and validation of basal upper tract urothelial carcinoma (UTUC) tumours across pathological stages. (A) Spatial transcriptomic maps of inferred malignant epithelial and mesenchymal signals in one non‐muscle‐invasive (NMI)‐basal section and two muscle‐invasive (MI)‐basal sections; colour intensity indicates relative signal strength. (B) Within‐section correlation matrices of inferred cell‐type signals for the same sections. (C) Representative immunohistochemical staining for CK5/6, GATA3, FAP and SPP1 in NMI‐basal and MI‐basal tumours. Scale bars, 200 µm.

To clarify the cellular basis of these signals, we performed scRNA‐seq and, after quality control and marker‐based annotation, identified major epithelial, lymphoid (CD4^+^ T cells, CD8^+^ T cells, B cells and NK cells), myeloid (monocytes and macrophages, dendritic cells [DCs], neutrophils and mast cells), mesenchymal and endothelial lineages (Figures 1D,E and 2A,B). MI samples showed the highest proportion of malignant epithelial cells, whereas adjacent tissues were enriched in normal epithelium and CD8^+^ T cells; in contrast, myeloid and endothelial cells (ECs) were expanded in tumours (Figures 1F, S1D and S2C). We integrated single‐cell data with genome‐wide association study (GWAS) risk signals and found significant associations between UTUC genetic risk and malignant epithelial cells, monocytes, macrophages, neutrophils, DCs, mesenchymal cells and ECs (Figure S3A–C).

We next assessed whether the immune‒stromal enrichment of the basal subtype was spatially preserved across stages. ST analysis showed consistent enrichment of immune and stromal cell populations in both NMI and MI basal tumours (Figures 2A,B and S4A–D). Immunohistochemistry (IHC) further confirmed elevated SPP1 and FAP expression in basal tumours regardless of stage (Figure 2C), two markers commonly associated with myeloid‐mediated immunosuppression and activated stromal fibroblasts, respectively.^13^, ^14^ Together, these findings indicate that basal UTUC maintains an immune‒stromal‐enriched state across pathological stages, with molecular subtype serving as the dominant organiser of the TME.

### 2.2 Developmental and spatial heterogeneity of malignant epithelial cells in UTUC

After inferCNV‐based identification, re‐clustering resolved five malignant epithelial subclusters with distinct marker profiles (Figures S5A,B,D and S6A). An alternative‐reference inferCNV analysis using within‐sample immune cells, stromal cells and ECs yielded concordant malignant‐cell assignments, epithelial subcluster structure, marker profiles and Hallmark pathway activities (Figure S13). Cancer_c0 showed the greatest developmental potential, whereas Cancer_c3 exhibited distinct trajectory and regulon profiles, enrichment for hypoxia, glycolysis and angiogenesis‐related programs, preferential localisation to the tumour side of the inferred boundary in MI‐basal sections, and an association with poorer disease‐specific survival (DSS) (Figures S5C–J and S6B–F). Across 22 external BCa sections, both Cancer_c0 and Cancer_c3 scores increased towards the basal end of the epithelial basal‒luminal continuum (Figure S6G); in the Japan‐UTUC cohort, the Cancer_c3 score remained associated with muscle invasion after adjustment for age and sex (Figure S6H).

### 2.3 Stage‐associated spatial remodelling of the myeloid compartment

**FIGURE 3:**
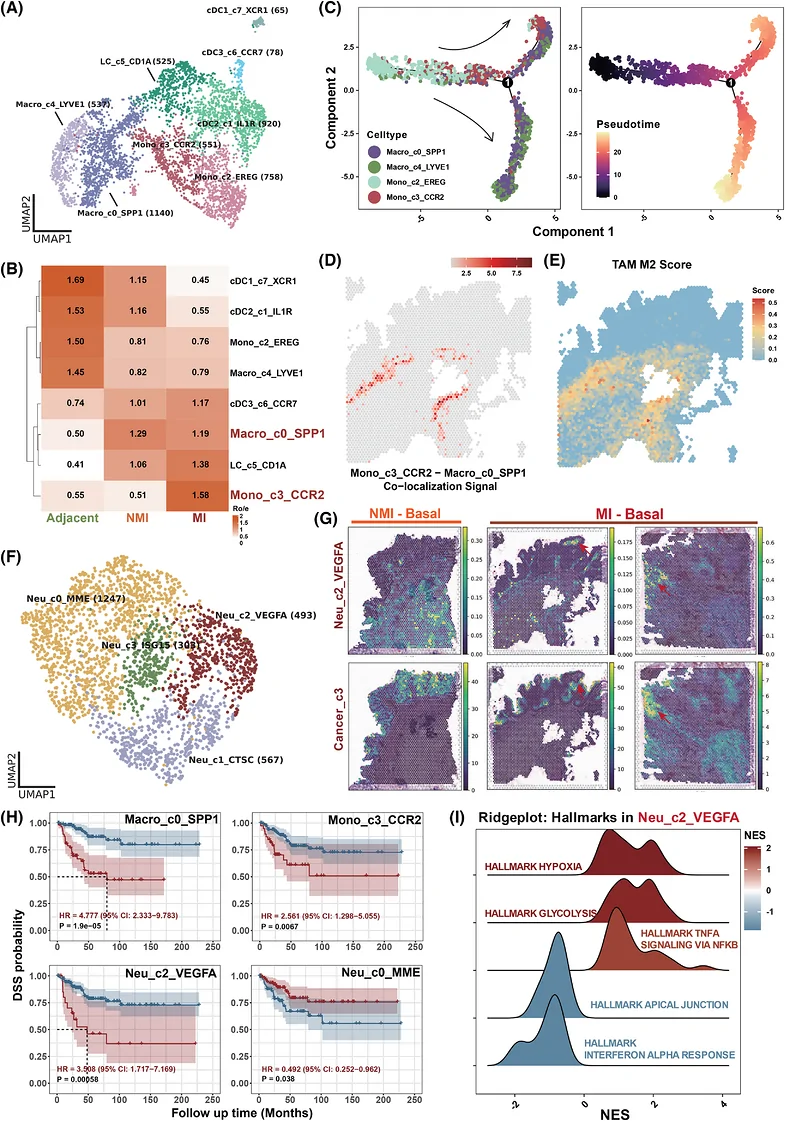
Myeloid‐state heterogeneity and spatial remodelling across upper tract urothelial carcinoma (UTUC) stages. (A) UMAP of re‐clustered myeloid cells showing macrophage, monocyte, dendritic cell and related subclusters. (B) *R*_o/e_ analysis of myeloid‐subcluster abundance in adjacent tissues, non‐muscle‐invasive (NMI) tumours and muscle‐invasive (MI) tumours. (C) Monocyte‐to‐macrophage trajectory coloured by cell state (left) and pseudotime (right). (D) Spatial co‐localisation signal for Mono_c3_CCR2 and Macro_c0_SPP1. (E) Spatial distribution of the TAM M2 score. (F) UMAP of neutrophil subclusters. (G) Spatial distributions of Neu_c2_VEGFA and Cancer_c3 in one NMI‐basal and two MI‐basal sections. (H) Kaplan‒Meier analyses of disease‐specific survival (DSS) according to representative myeloid‐state scores. (I) Hallmark gene‐set enrichment profile of Neu_c2_VEGFA.

Re‐clustering and trajectory analysis delineated a monocyte‐to‐macrophage continuum from Mono_c3_CCR2 monocytes to Macro_c0_SPP1 macrophages (hereafter termed SPP1^+^ TAMs) (Figures 3A–C and S7A–C). Compared with the NMI‐basal section, the two states showed greater spatial co‐enrichment in MI‐basal sections and overlapped with elevated M2‐polarisation signals; both were associated with poorer survival (Figures 3D–E,H and S7D).

Neutrophil analysis identified Neu_c0_MME as a favourable state and Neu_c2_VEGFA as a poor‐prognosis state, hereafter termed VEGFA^+^ TANs (Figure 3F–H). In MI‐basal sections, VEGFA^+^ TANs showed a stronger spatial association with Cancer_c3 than in the NMI‐basal section and exhibited enrichment of hypoxia, glycolysis and inflammatory‐response programs (Figure 3G,I). Plasmacytoid DCs and CCR7‐high cDCs were likewise associated with unfavourable prognosis (Figure S7E). Together, these findings nominate SPP1^+^ TAMs and VEGFA^+^ TANs as high‐risk myeloid states linked to the myeloid‒stromal and angiogenic programs examined below.

### 2.4 Functional heterogeneity of lymphoid cells in UTUC

Re‐clustering of lymphoid cells identified B cell, CD4^+^ T cell, CD8^+^ T cell, NK cell and plasma‐cell subsets with distinct functional and prognostic associations. Tumour‐enriched cytotoxic/exhausted T‐cell states, FOXP3^+^ Tregs and spatial T‐cell inhibition/Treg programs were evident in basal tumours, whereas TLS‐like regions were observed in NMI samples (Figures S8 and S9). Together, these data indicate that basal UTUC contains both immune‐activated and immunosuppressive lymphoid states.

### 2.5 Stromal‒endothelial heterogeneity and remodelling in UTUC

**FIGURE 4:**
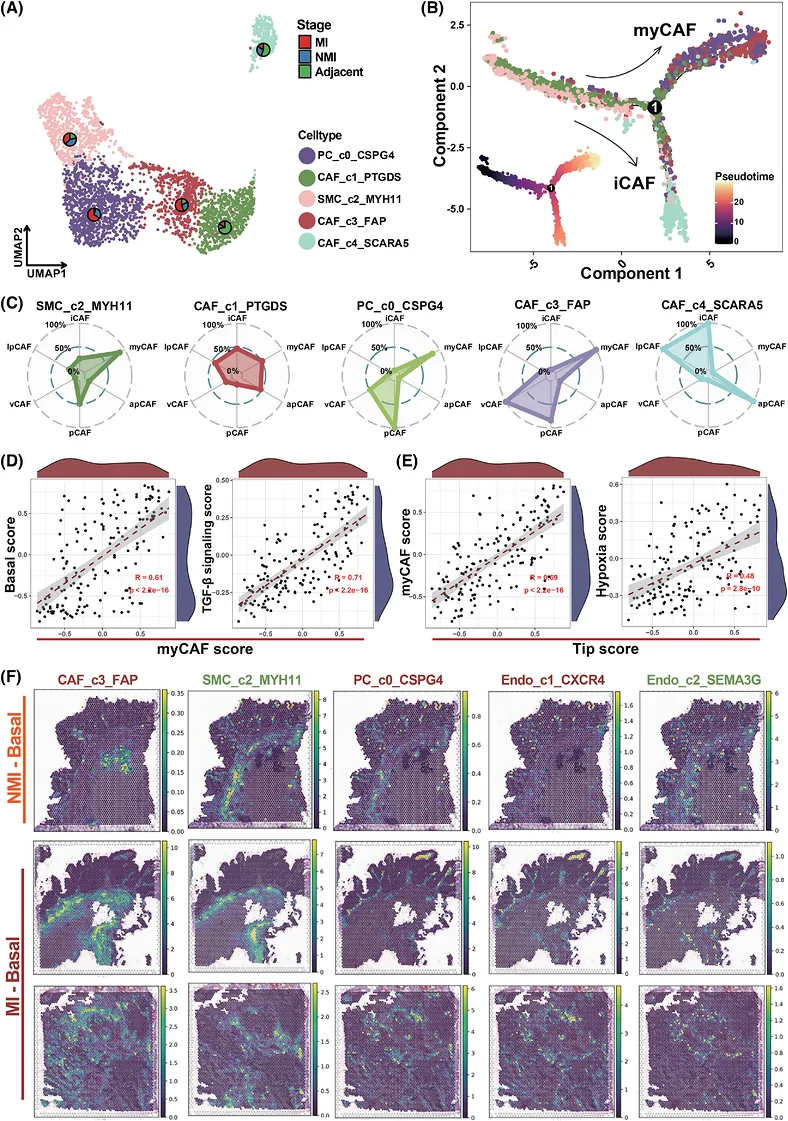
Functional states and spatial distribution of stromal and endothelial subclusters in upper tract urothelial carcinoma (UTUC). (A) UMAP of mesenchymal subclusters; inset pie charts show their relative stage composition. (B) Pseudotime trajectory showing branching towards inflammatory CAF (iCAF) and myofibroblastic CAF (myCAF) states. (C) Radar plots of CAF‐state signatures across mesenchymal subclusters. (D) Associations of the myCAF score with the basal score (left) and transforming growth factor beta (TGF‐β) signalling score (right). (E) Associations of the tip‐cell score with the myCAF score (left) and hypoxia score (right). (F) Spatial distributions of CAF_c3_FAP, SMC_c2_MYH11, PC_c0_CSPG4, Endo_c1_CXCR4 and Endo_c2_SEMA3G in one non‐muscle‐invasive (NMI)‐basal and two muscle‐invasive (MI)‐basal sections.

Mesenchymal re‐clustering and trajectory analysis resolved five subclusters, including CAF_c3_FAP cells (hereafter termed FAP^+^ myofibroblastic cancer‐associated fibroblast (CAF) [myCAFs]) (Figures 4A and 10A). FAP^+^ myCAFs displayed a myofibroblastic, matrix‐producing phenotype with enrichment of EMT and glycolysis programs (Figures 4B,C and S10B,C). The myCAF score correlated positively with the basal score and transforming growth factor beta (TGF‐β) signalling activity (Figure 4D).

Endothelial analysis identified Endo_c1_CXCR4 cells (hereafter termed CXCR4^+^ tip ECs) as a tip‐like state (Figure S10D,E). CXCR4^+^ tip ECs were enriched in hypoxia‐related and IL6‒JAK‒STAT3 signalling (Figure S10F), and the tip score correlated positively with the myCAF and hypoxia scores (Figure 4E). Spatial mapping showed that FAP^+^ myCAFs and CXCR4^+^ tip ECs were more prominent in the MI‐basal sections, whereas SMCs and arterial ECs were more prominent in the NMI‐basal section (Figure 4F). FAP^+^ myCAFs and CXCR4^+^ tip ECs were associated with poorer prognosis, whereas arterial ECs were associated with more favourable outcomes (Figure S10G). These findings prioritised FAP^+^ myCAFs and CXCR4^+^ tip ECs for subsequent analyses of the myeloid‒stromal and angiogenic programs, respectively.

### 2.6 SPP1^+^ TAMs‒FAP^+^ myCAF coupling in an immunosuppressive stromal boundary of MI‐basal UTUC

**FIGURE 5:**
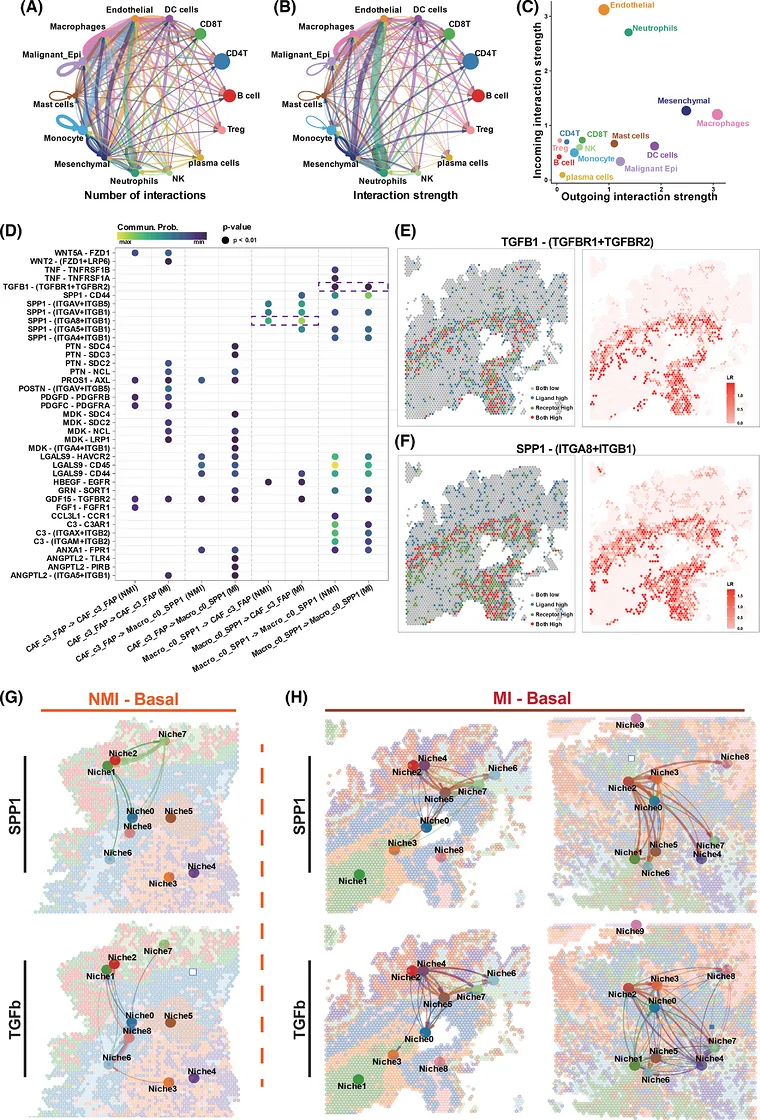
Global cell‒cell communication and spatial mapping of SPP1^+^ TAM‒FAP^+^ myofibroblastic CAF (myCAF) signalling in basal upper tract urothelial carcinoma (UTUC). (A) Number of inferred ligand‒receptor interactions among tumour‐microenvironment cell types. (B) Aggregate interaction strength among cell types. (C) Outgoing versus incoming communication strength for each cell type. (D) Selected ligand‒receptor pairs within and between Macro_c0_SPP1 (SPP1^+^ TAMs) and CAF_c3_FAP (FAP^+^ myCAFs) in non‐muscle‐invasive (NMI)‐basal and muscle‐invasive (MI)‐basal sections. (E and F) Spatial maps of TGFβ1‒(TGFBR1+TGFBR2) (E) and SPP1‒(ITGA8+ITGB1) (F), showing categorical ligand/receptor co‐expression (left) and the continuous ligand‒receptor signal (right). (G and H) Spatial communication networks for SPP1 and TGFβ1 in the NMI‐basal section (G) and the two MI‐basal sections (H).

**FIGURE 6:**
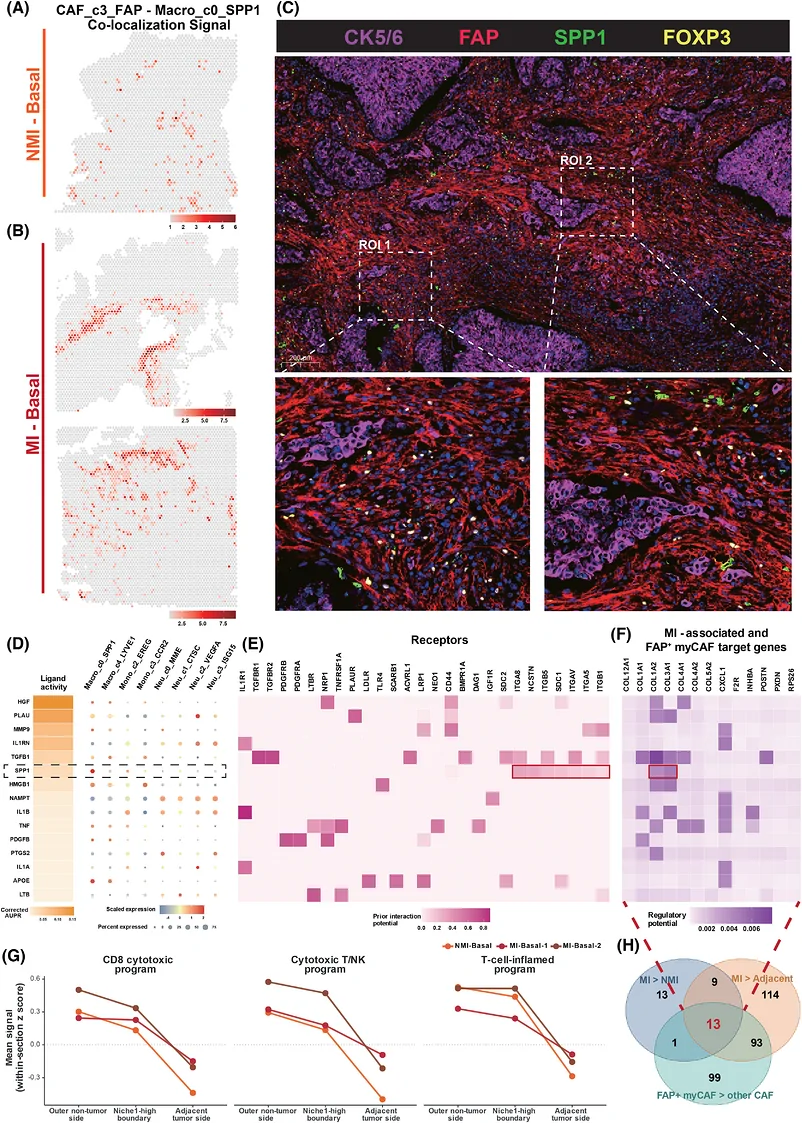
Spatial organisation and receiver programs of the SPP1^+^ TAM‒FAP^+^ myofibroblastic CAF (myCAF) niche in basal upper tract urothelial carcinoma (UTUC). (A and B) Abundance‐based spatial co‐localisation of SPP1^+^ TAM and FAP^+^ myCAF signals in the non‐muscle‐invasive (NMI)‐basal section (A) and the two muscle‐invasive (MI)‐basal sections (B). (C) Multiplex immunofluorescence in an MI‐basal tumour showing CK5/6 (purple), FAP (red), SPP1 (green) and FOXP3 (yellow), with enlarged views of the indicated regions; scale bars are shown. (D) NicheNet ligand‐activity ranking and expression of candidate ligands across myeloid sender states. (E) Prior interaction potential between candidate ligands and receptors expressed by FAP^+^ myCAFs. (F) Regulatory potential of candidate ligands for the 13‐gene, MI‐associated FAP^+^ myCAF receiver program. (G) Within‐section mean *z*‐scores for CD8‐cytotoxic, cytotoxic T/NK and T‐cell‐inflamed programs across the outer non‐tumour side, the Niche1‐high stromal boundary and the adjacent tumour side; lines connect regions from the same section. (H) Overlap of receiver genes increased in MI versus NMI, MI versus adjacent tissue, and FAP^+^ myCAFs versus other CAFs.

CellChat analysis characterised the global UTUC TME communication network by interaction number and strength, identifying ECs, neutrophils, mesenchymal cells and macrophages as major communication hubs (Figure 5A–C). A prominent myeloid‒stromal axis emerged between SPP1^+^ TAMs and FAP^+^ myCAFs, defining Niche1 through reciprocal signals related to matrix remodelling, cell adhesion and immune regulation, including TGFβ1‒TGFBR1/2 and SPP1‒integrin interactions (Figure 5D). Across complementary spatial dimensions, these ligand‒receptor pairs showed coordinated tissue distributions (Figure 5E,F), SPP1‐ and TGFβ1‐associated communication networks extended over broader regions in MI‐basal sections (Figure 5G,H), and SPP1^+^ TAMs and FAP^+^ myCAFs exhibited more extensive boundary‐associated co‐localisation in MI‐basal than in NMI‐basal tumours (Figure 6A,B). Multiplex immunofluorescence (mIF) further supported this organisation, showing FAP‐rich stroma accompanied by SPP1^+^ and FOXP3^+^ signals around CK5/6^+^ tumour epithelium (Figure 6C). NicheNet analysis placed SPP1 among the leading macrophage‐derived ligands and linked the receiver response of FAP^+^ myCAFs predominantly to ECM assembly and collagen remodelling in association with muscle invasion (Figure 6D–F,H). Across all three basal sections, CD8‐cytotoxic, cytotoxic T/NK and T‐cell‐inflamed scores progressively declined from the outer non‐tumour side across Niche1‐high stromal boundaries towards the adjacent tumour compartment (Figure 6G), characterising Niche1 as an MI‐associated, matrix‒remodelling stromal interface accompanied by reduced effector immune activity towards the tumour.

### 2.7 External BCa support for the SPP1‐associated myeloid‒stromal program

**FIGURE 7:**
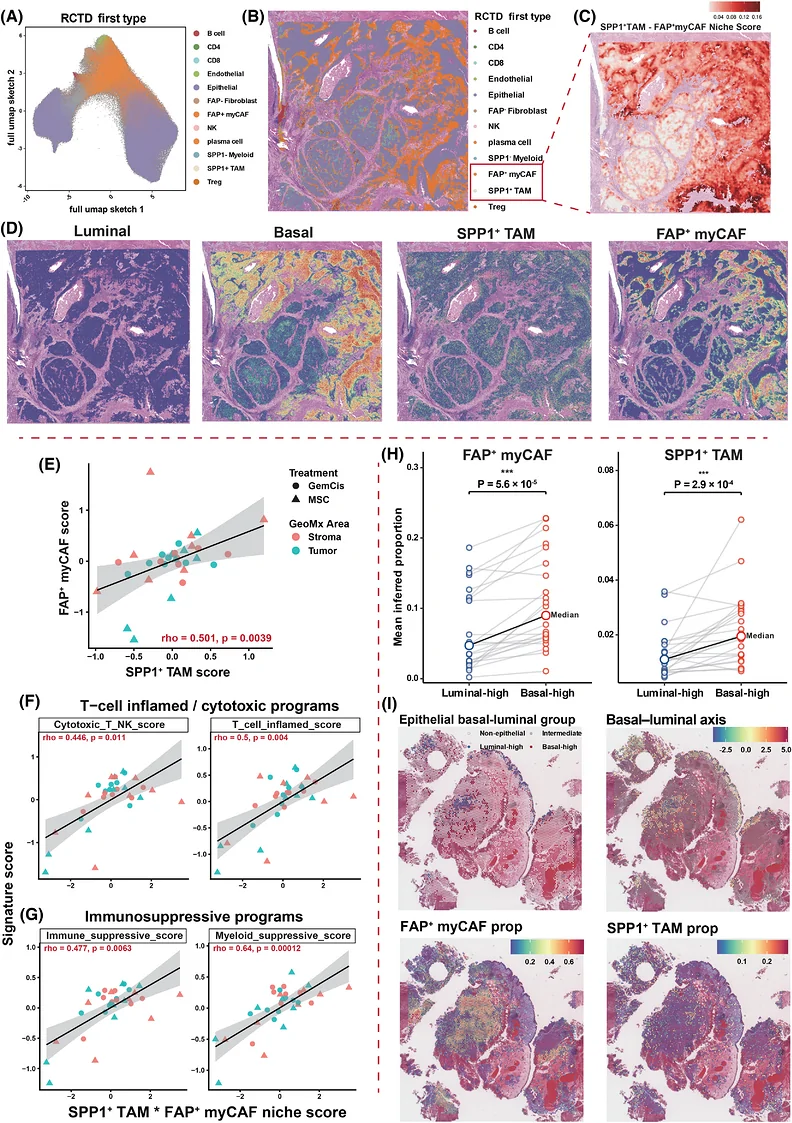
External bladder urothelial carcinoma datasets support the SPP1^+^ TAM‒FAP^+^ myofibroblastic CAF (myCAF) program. Visium HD analysis (GSE299573): (A) RCTD‐based UMAP sketch of major inferred cell types in a public bladder urothelial carcinoma section. (B) Representative RCTD cell‐type map overlaid on tissue morphology. (C) Spatial distribution of the composite SPP1^+^ TAM‒FAP^+^ myCAF program score in the indicated region. (D) Spatial maps of luminal, basal, SPP1^+^ TAM and FAP^+^ myCAF signals in the same section. GeoMx analysis (GSE296955): (E) association between SPP1^+^ TAM and FAP^+^ myCAF scores across tumour and stromal regions and treatment groups. (F and G) Associations of the composite score with T‐cell‐inflamed/cytotoxic programs (F) and immune‐suppressive/myeloid‐suppressive programs (G). Multi‐sample paired Visium analysis (GSE319536): (H) paired comparison of inferred FAP^+^ myCAF and SPP1^+^ TAM proportions between luminal‐high and basal‐high regions. (I) Spatial maps of epithelial basal‒luminal group assignment, the basal‒luminal axis, and inferred FAP^+^ myCAF and SPP1^+^ TAM proportions. Statistical values are shown in the corresponding panels.

External validation showed that the SPP1‐associated immune‒stromal state of basal UTUC was preserved across pathological stages in BCa (Figure S11A–E and Table S4). Independent single‐cell datasets further resolved its two cellular components, SPP1^+^ TAMs and FAP^+^ myCAFs (Figure S12). This conserved association with the basal phenotype extended to spatial organisation, as the two populations co‐enriched near basal‐high epithelium and were more abundant in basal‐high than in luminal‐high regions (Figure 7A–D,H,I). Their joint program was associated with both inflamed/cytotoxic and immune/myeloid‐suppressive signals, recapitulating the immune‐active yet suppressive context of basal UTUC (Figure 7E–G). These findings establish the SPP1^+^ TAM‒FAP^+^ myCAF program as a conserved basal‐associated myeloid‒stromal feature across UC contexts.

### 2.8 Epithelial‐intrinsic functional effects of SPP1 loss in a UC model

**FIGURE 8:**
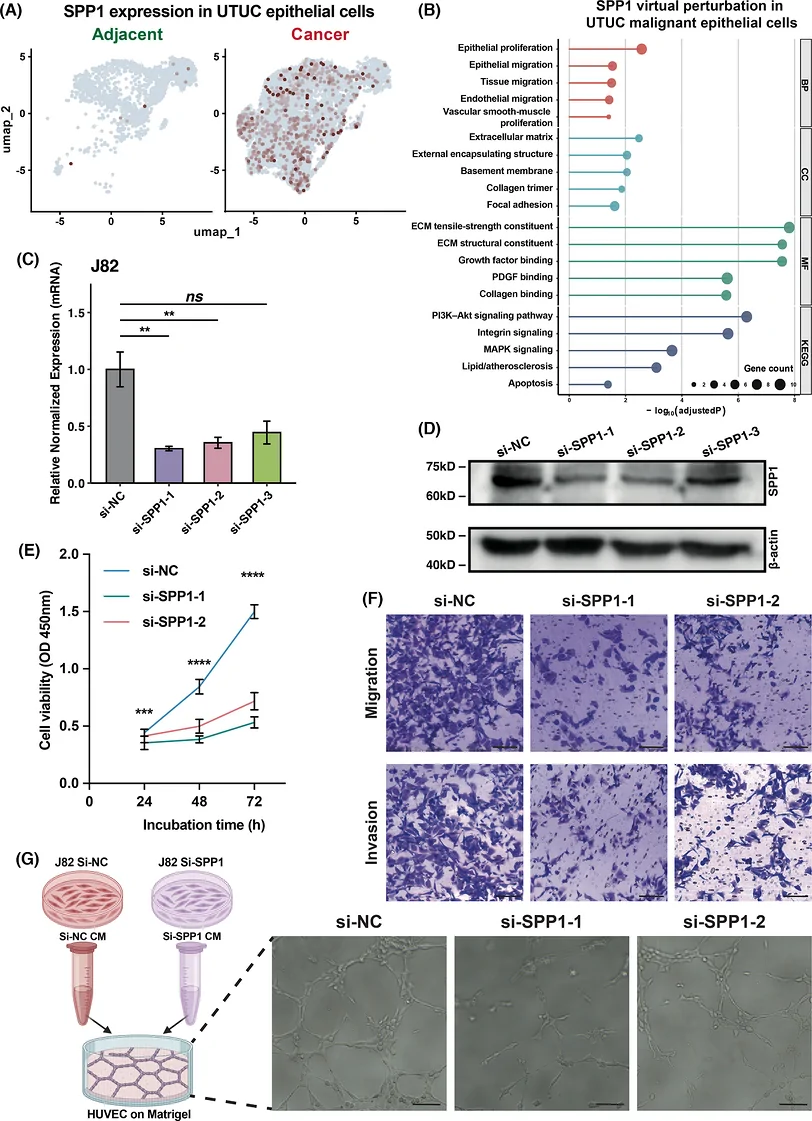
Epithelial SPP1 expression and functional effects of SPP1 knockdown in a urothelial carcinoma model. (A) SPP1 expression in adjacent and malignant epithelial cells from the upper tract urothelial carcinoma (UTUC) single‐cell cohort. (B) Gene Ontology biological process, cellular component and molecular function terms and Kyoto Encyclopedia of Genes and Genomes (KEGG) pathways associated with virtual SPP1 perturbation in UTUC malignant epithelial cells. (C) qRT‐PCR screening of three SPP1‐targeting siRNAs in J82 cells. (D) Western blot assessment of SPP1 protein after siRNA transfection, with β‐actin as the loading control. (E) Cell Counting Kit‐8 (CCK‐8) analysis of J82 cell viability after SPP1 knockdown with si‐SPP1‐1 or si‐SPP1‐2. (F) Representative Transwell migration and invasion images. (G) Experimental workflow and representative human umbilical vein endothelial cell (HUVEC) tube‐formation images after exposure to conditioned medium from control or SPP1‐knockdown J82 cells. Scale bars in F and G, 100 µm. ns, not significant; ^**^ *p* < .01; ^***^ *p* < .001; ^****^ *p* < .0001.

SPP1 was more broadly detected in malignant than in adjacent epithelial cells, prompting us to examine its epithelial‐intrinsic function beyond the myeloid‒stromal compartment (Figure 8A). Virtual SPP1 knockout in malignant epithelial cells implicated proliferation, migration, ECM organisation and adhesion‐related signalling (Figure 8B). Consistent with these predictions, two independent siRNAs efficiently depleted SPP1 in J82 cells and reduced cell viability, migration and invasion (Figure 8C–F). Conditioned medium (CM) from SPP1‐depleted J82 cells also impaired human umbilical vein endothelial cell (HUVEC) tube formation (Figure 8G). Together, these findings support an epithelial‐intrinsic role for SPP1 in malignant behaviour and angiogenesis‐associated activity in a UC model.

### 2.9 MI‐associated VEGFA^+^ TAN‒CXCR4^+^ tip EC angiogenic program

**FIGURE 9:**
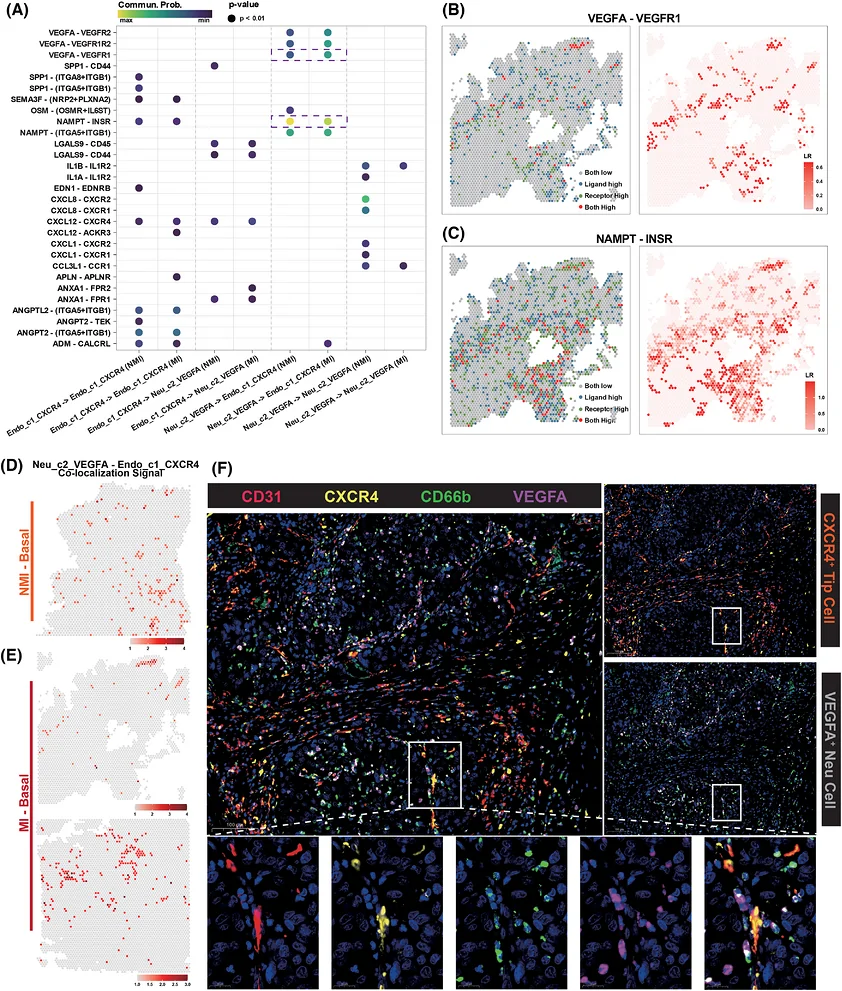
Spatial coupling and ligand‒receptor interactions between CXCR4^+^ tip endothelial cells (ECs) and VEGFA^+^ TAN in basal upper tract urothelial carcinoma (UTUC). (A) Selected ligand‒receptor interactions within and between Neu_c2_VEGFA (VEGFA^+^ TANs) and Endo_c1_CXCR4 (CXCR4^+^ tip ECs) in non‐muscle‐invasive (NMI)‐basal and muscle‐invasive (MI)‐basal sections. (B and C) Spatial maps of VEGFA‒VEGFR1 (B) and NAMPT‒INSR (C), showing categorical ligand/receptor co‐expression (left) and the continuous ligand‒receptor signal (right). (D‒E) Abundance‐based Neu_c2_VEGFA‒Endo_c1_CXCR4 co‐localisation signals in the NMI‐basal section (D) and the two MI‐basal sections (E). (F) Multiplex immunofluorescence in an MI‐basal tumour showing CD31 (red), CXCR4 (yellow), CD66b (green) and VEGFA (purple), with enlarged views of the indicated regions; scale bars are shown.

Within the same global communication landscape, a distinct neutrophil‒endothelial branch linked VEGFA^+^ TANs with CXCR4^+^ tip ECs, defining the angiogenic program Niche2 (Figure 5A–C). Candidate interactions within Niche2 converged on vascular remodelling, hypoxia‐associated metabolic adaptation and inflammatory chemotaxis, with VEGFA‒VEGFR1 and NAMPT‒INSR selected as representative axes for spatial validation (Figure 9A). Spatial mapping showed coordinated distributions of both ligand‒receptor signals, while abundance‐based maps revealed more extensive co‐localisation of VEGFA^+^ TANs and CXCR4^+^ tip ECs in MI‐basal than in NMI‐basal sections (Figure 9B–E). mIF further localised CD66b^+^VEGFA^+^ neutrophils and CD31^+^CXCR4^+^ ECs within the same MI‐basal tissue regions (Figure 9F).

### 2.10 Niche1 enrichment at tumour boundaries co‐occurs with basal‐polarised epithelium and is associated with adverse prognosis

**FIGURE 10:**
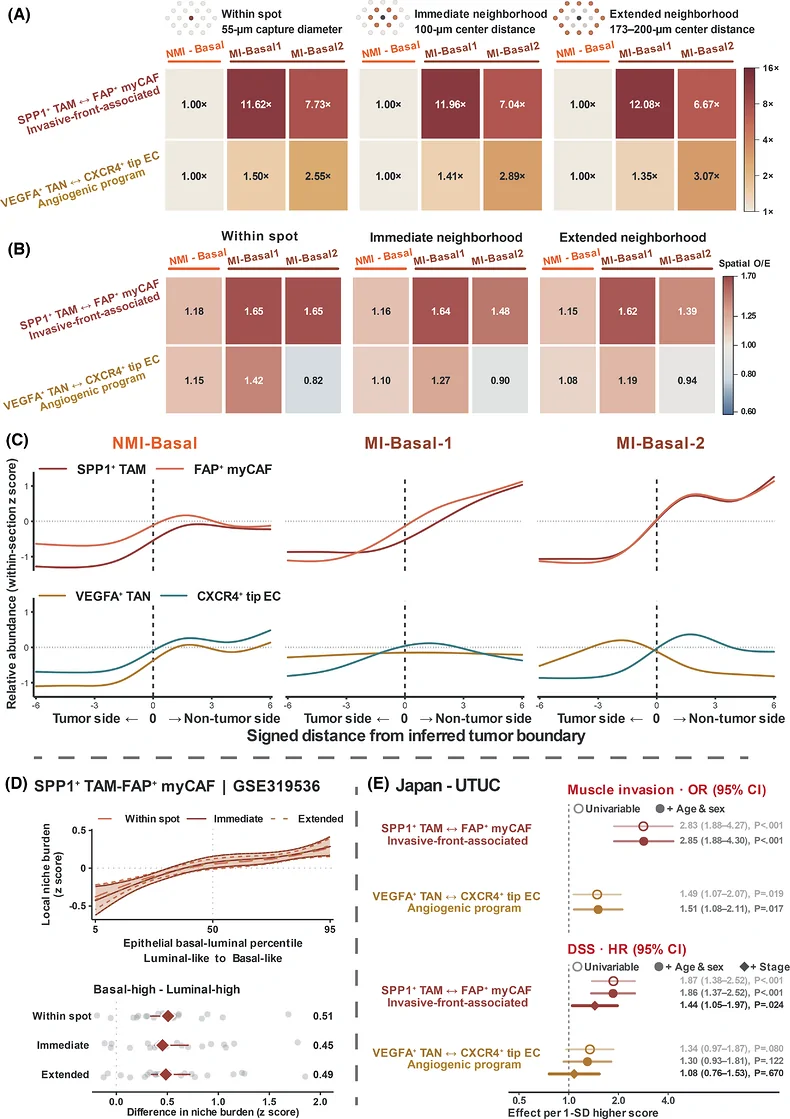
Multiscale spatial and clinical comparison of two microenvironmental programs in basal upper tract urothelial carcinoma (UTUC). (A) Abundance‐weighted pair burden for the SPP1^+^ TAM‒FAP^+^ myofibroblastic CAF (myCAF) and VEGFA^+^ TAN‒CXCR4^+^ tip endothelial cell (EC) programs at within‐spot, immediate‐neighbourhood and extended‐neighbourhood scales; values are fold changes relative to the non‐muscle‐invasive (NMI)‐basal section at the same scale. (B) Within‐section observed‐to‐expected spatial enrichment (O/E) based on 500 positional permutations; O/E > 1 indicates greater co‐enrichment than expected by chance. (C) Section‐specific standardised abundance profiles across the inferred tumour boundary; negative and positive distances indicate the tumour and non‐tumour sides, respectively. (D) Association of the SPP1^+^ TAM‒FAP^+^ myCAF burden with the epithelial luminal‐to‐basal axis in 22 MIBC Visium sections from GSE319536. Curves show the equal‐weight section summary; the lower plot shows section‐specific basal‐high minus luminal‐high differences. (E) Odds ratios for muscle invasion and hazard ratios for disease‐specific survival (DSS) per 1‐SD higher bulk‐derived cellular‐program score, with 95% confidence intervals, in the Japan‐UTUC cohort. Models show univariable and age‐ and sex‐adjusted estimates, with additional stage adjustment for DSS.

Across three spatial scales, both niches showed higher abundance‐weighted pair burdens in MI‐basal than in NMI‐basal sections, with a substantially greater increase for Niche1 (Figure 10A). Niche1 further showed reproducible, non‐random co‐enrichment at the tumour boundary, whereas Niche2 exhibited a more heterogeneous spatial pattern across sections (Figures 10B,C and S14A). This boundary organisation of Niche1 was associated with ECM assembly and collagen organisation, whereas Niche2 was associated with hypoxia and angiogenesis (Figure S14B,C). Across 22 external MIBC sections, Niche1 burden increased towards the basal end of the epithelial luminal‐to‐basal continuum (Figure 10D). In the Japan‐UTUC cohort, both niches were associated with muscle invasion after adjustment for age and sex, but only Niche1 remained associated with DSS after further adjustment for stage (Figure 10E).

### 2.11 SPP1 links the basal phenotype to adverse prognosis in UTUC

**FIGURE 11:**
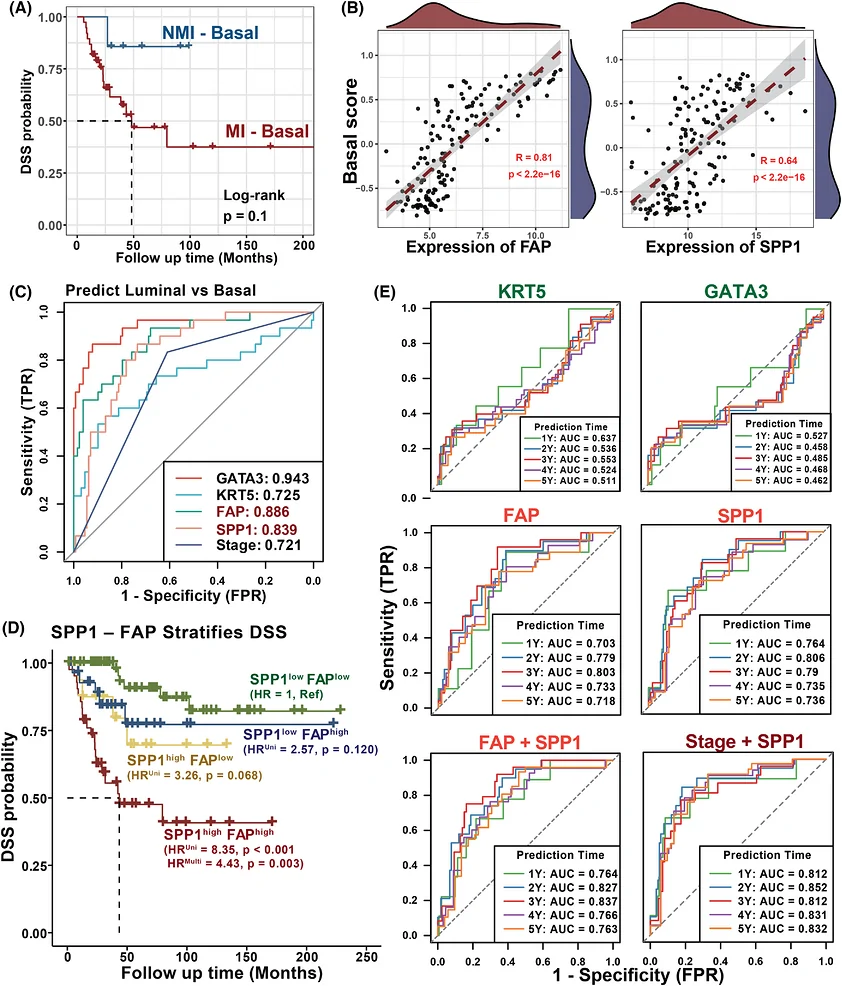
Clinical stratification and subtype‐discrimination performance of SPP1 and FAP in upper tract urothelial carcinoma (UTUC). (A) Kaplan‒Meier analysis of disease‐specific survival (DSS) in basal non‐muscle‐invasive (NMI) versus basal muscle‐invasive (MI) tumours. (B) Associations of the basal score with FAP (left) and SPP1 (right) expression. (C) Receiver operating characteristic curves for luminal‒basal discrimination using GATA3, KRT5, FAP, SPP1 or pathological stage. (D) DSS stratification by joint SPP1/FAP expression, with each marker dichotomised as high or low; HRUni and HRMulti denote hazard ratios from univariable and stage‐adjusted Cox models, respectively. (E) Time‐dependent receiver operating characteristic curves for 1‐ to 5‐year DSS prediction using KRT5, GATA3, FAP, SPP1, FAP + SPP1 or stage + SPP1.

MI‐basal patients showed a nonsignificant trend towards poorer DSS than NMI‐basal patients, while only Niche1 remained associated with DSS after stage adjustment (Figures 10E and 11A). We therefore evaluated the clinical stratification value of SPP1 and FAP in basal UTUC. Both markers tracked the basal phenotype and distinguished basal from luminal tumours comparably to GATA3 and KRT5 (Figure 11B,C). Joint SPP1/FAP stratification identified the SPP1‐high/FAP‐high group as having the poorest DSS, and both markers achieved 5‐year AUCs above .7, compared with values near .5 for GATA3 and KRT5 (Figure 11D,E). SPP1 remained associated with DSS after stage adjustment (hazard ratio [HR] = 1.17, 95% confidence interval [CI] 1.01‒1.35, *p* = .036; Table S2). Among the evaluated models, combining SPP1 with stage yielded the highest 5‐year area under the receiver operating characteristic curve (AUC), exceeding .8 (Figure 11E). Phenome‐wide Mendelian randomisation identified no widespread systemic disease‐risk signal associated with SPP1 (Table S3).

## 3 DISCUSSION

Our study identifies an immune‒stromal‐enriched microenvironment as a largely stage‐independent feature of basal UTUC. Within this subtype, muscle invasion was accompanied by spatial reorganisation into two programs. Niche1 was a reproducible SPP1^+^ TAM‒FAP^+^ myCAF boundary program, whereas Niche2 was a more heterogeneous VEGFA^+^ TAN‒CXCR4^+^ tip EC angiogenic program.

Analogous SPP1^+^ TAM‒FAP^+^ myCAF barriers have been reported at invasive fronts in colorectal cancer and non‐small cell lung cancer, where they are associated with immune exclusion and adverse outcomes.^15^, ^16^ In MI‐basal UTUC, increased multiscale pair burden, preferential enrichment at tumour boundaries, and a stepwise decline in effector immune scores towards the tumour collectively support a comparable spatial architecture. Within Niche1‐enriched tumour boundaries, spatial analysis highlighted candidate TGFβ1‒TGFBR1/2 and SPP1‒ITGA8/ITGB1 signalling axes, consistent with fibroblast activation, matrix remodelling and myeloid‐mediated immune suppression.^17^, ^18^ Complementary NicheNet analysis placed SPP1 within a broader TAM‐to‐FAP^+^ myCAF ligand program associated with muscle invasion, with predicted receiver‐cell targets converging on ECM assembly and collagen remodelling.

External BCa single‐cell datasets also identified distinct SPP1^+^ TAM and FAP^+^ myCAF populations. Yu et al. defined a continuous luminal‐to‐basal epithelial axis across 22 MIBC tumours, with basal‐like states preferentially localised towards invasive margins.^19^ In our reanalysis, Niche1 burden increased towards the basal pole, indicating that basal epithelial organisation is accompanied by a distinct myeloid‒stromal program. At higher spatial resolution, Visium HD analysis of a bladder tumour with basal/squamous differentiation showed local co‐enrichment of inferred SPP1^+^ TAM and FAP^+^ myCAF signals near basal‐high epithelium. GeoMx data further suggest that Niche1 marks an inflamed yet immunosuppressive microenvironment, in which cytotoxic immune programs coexist with myeloid suppression. These external findings support a model in which basal‐like tumour epithelium is spatially coupled with an ECM remodelling, immunosuppressive myeloid‒stromal program at invasive tumour boundaries.

By contrast, Niche2 resembles an angiogenic program described in triple‐negative breast cancer, where VEGFA^+^ TANs interact with APLN^+^ tip ECs within hypoxic regions to establish pro‐angiogenic niches.^20^ The VEGFA^+^ TAN‒CXCR4^+^ tip EC pair in basal UTUC may represent a related neutrophil‒endothelial program. Unlike Niche1, Niche2 could not be directly evaluated in external BCa scRNA‐seq datasets because these datasets lacked a discrete neutrophil population suitable for state‐level analysis. This may partly reflect the low RNA content and processing sensitivity of neutrophils, which can lead to their under‐recovery or exclusion by standard quality‐control filters.^21^ Niche2 also showed a section‐dependent spatial pattern; in the Japan‐UTUC cohort, the corresponding angiogenic program was associated with muscle invasion but not with DSS after stage adjustment. Niche2 therefore remains an exploratory, MI‐associated angiogenic program requiring validation in larger UTUC cohorts using neutrophil‐preserving single‐cell workflows and high‐resolution spatial profiling.

Taken together, basal UTUC combines immune activation and suppression: effector‐cell enrichment coexists with exhausted CD8^+^ T cells, CD4^+^ Tregs and increased checkpoint signalling. Previous studies have associated immune‐rich, high‐risk UTUC states with potential ICB responsiveness and shown that benefit varies across UC molecular subtypes.^11^, ^22^ Our spatial analysis provides a potential explanation: in MI‐basal tumours, this immune‐active state is embedded within an ECM remodelling, myeloid‐suppressive Niche1 boundary and a hypoxia‐associated angiogenic Niche2. The boundary‐associated effector gradient suggests that the SPP1^+^ TAM‒FAP^+^ myCAF program may restrict effector‐cell access, whereas the VEGFA^+^ TAN‒CXCR4^+^ tip EC program may impose an additional angiogenic constraint.^16^, ^23^, ^24^ Thus, immune enrichment does not necessarily translate into effective antitumour immunity. Coupled with the elevated EGFR signalling towards the basal pole reported by Yu et al., our findings suggest that the therapeutic vulnerabilities of basal tumours span both epithelial lineage programs and the surrounding microenvironment.^19^ Combining ICB with approaches targeting basal‐associated epithelial signalling and the accompanying myeloid‒stromal or angiogenic barriers therefore warrants investigation. The TLS‐like regions observed more prominently in the NMI sample provide a complementary example: effective antitumour immunity may depend not only on immune cell accumulation but also on spatial organisation and maturation. In ccRCC, tumour‐proximal TLS were associated with favourable outcomes, whereas tumour‐distal TLS, which were predominantly immature, were linked to Treg and PD‐L1^+^ TAM enrichment and poorer outcomes.^25^

SPP1 links molecular subtype, spatial niche remodelling, and clinical outcome in UTUC. Within Niche1, SPP1‐associated TAM‒myCAF signalling may couple myeloid suppression to fibroblast activation and ECM deposition at the tumour boundary. Epithelial SPP1 also supports tumour‐cell viability, migration and invasion. For the conditioned‐medium experiment, recombinant SPP1 rescue is needed to determine whether the reduction in HUVEC tube formation specifically reflects loss of secreted SPP1. Separately, a macrophage‐specific SPP1 knockout model could test whether macrophage‐derived SPP1 is required to sustain Niche1 in vivo. This model could further assess whether macrophage‐specific SPP1 loss reduces ECM deposition and myeloid suppression, restores effector‐cell access, and improves responses to subsequent lineage‐matched therapy. Clinically, SPP1 distinguished the basal phenotype with performance comparable to established subtype markers and provided prognostic information beyond pathological stage. PheW‐MR revealed no broad systemic disease‐risk signals, providing preliminary genetic context for the therapeutic evaluation of SPP1.

Overall, our findings suggest that basal UTUC harbours a largely stage‐independent immune‒stromal background, while muscle invasion is associated with its spatial reorganisation into a reproducible SPP1‐associated boundary program and a more heterogeneous angiogenic program. Single‐cell‐resolution spatial profiling could map these programs more precisely by resolving cell‒cell distances and neighbourhood composition within the UTUC TME.^26^ Expanding such profiling to larger, multi‐patient UTUC cohorts would provide the sample size and diversity needed for robust model training and validation. Integrating these data with advanced computational approaches such as deep learning may then distinguish spatial relationships that recur across patients from patient‐specific variation and help evaluate their therapeutic relevance.^27^, ^28^

## 4 METHODS

### 4.1 Patient cohorts and data sources

Table S5 lists the datasets and public resources analysed in this study.

#### 4.1.1 Institutional UTUC cohort

This study was approved by the institutional review boards of the participating hospitals and conducted in accordance with the Declaration of Helsinki. Written informed consent was obtained from all patients prior to tissue collection. Tumour tissues from seven patients with primary UTUC were collected for scRNA‐seq; matched adjacent normal tissues were available for three of these patients. Molecular subtypes were defined as basal (CK5/6^+^/GATA3^−^) and luminal (CK5/6^−^/GATA3^+^) based on IHC. Three basal tumours were selected for ST analysis, including one NMI and two MI cases. All cases were confirmed by postoperative histopathology and independently reviewed by three experienced pathologists.

#### 4.1.2 External UTUC validation cohort

Bulk RNA‐seq data for an external Japanese UTUC cohort (*n* = 158) were obtained from the European Genome‒phenome Archive (EGA; accession number: EGAD00001007667).^3^ Raw reads were aligned to GRCh38 and quantified at the gene level, followed by variance‐stabilising transformation for downstream analyses.^29^ Molecular subtype annotations were derived from the original pathological classification reported in the corresponding study.

#### 4.1.3 GWAS data source

GWAS summary statistics for UTUC were obtained from the FinnGen R12 public release, including 256 cases and 378 749 controls.^30^

#### 4.1.4 Public BCa datasets for cross‐lineage validation

For cross‐lineage validation, we analysed bulk transcriptomic (TCGA‐BLCA and GSE32894), scRNA‐seq (GSE222315 and GSE267718), ST (GSE299573 and GSE319536) and GeoMx (GSE296955) datasets. Sample counts, comparators, access links and analytical roles are provided in Table S5; platform‐specific preprocessing is described below.

### 4.2 Single‐cell RNA sequencing

#### 4.2.1 Tissue dissociation and single‐cell suspension preparation

UTUC tumour specimens were processed for scRNA‐seq by NovelBio Bio‐Pharm Technology Co., Ltd. Before dissociation, fresh tissues were held in MACS Tissue Storage Solution (Miltenyi Biotec), rinsed in phosphate‐buffered saline (PBS), cut into small fragments, and digested for 30 min at 37°C with enzyme H (200 µL), enzyme R (100 µL) and enzyme A (25 µL) (Miltenyi Biotec). The resulting suspensions passed through a 70‐µm filter were centrifuged at 300 × *g* for 5 min, and underwent red blood cell lysis (Miltenyi Biotec). After washing, cells were resuspended in PBS containing .04% bovine serum albumin (BSA) and passed through a 35‐µm filter to generate single‐cell suspensions. Cell viability was assessed by AO/PI staining (Countstar).

#### 4.2.2 Library preparation and sequencing

Single‐cell libraries were constructed with the BD Rhapsody Whole Transcriptome Analysis (WTA) workflow following vendor instructions. Briefly, captured single cells received cell‐specific barcodes and unique molecular identifiers, after which reverse transcription, cDNA amplification and library construction were performed. Library quality was checked with Bioanalyser (Agilent) and Qubit (Thermo Fisher Scientific), and 150‐bp paired‐end reads were generated on an Illumina platform.

### 4.3 Spatial transcriptomics

#### 4.3.1 Tissue embedding and cryosectioning

Fresh tumour tissues were embedded in OCT compound (Sakura Finetek), snap‐frozen on dry ice and stored at −80°C. Tissue blocks were cryosectioned at 10 µm (Leica CM1950) and mounted onto Visium Spatial Gene Expression slides (10× Genomics).

#### 4.3.2 Haematoxylin and eosin staining and imaging

Sections were equilibrated at 37°C for 1 min and fixed in pre‐chilled methanol (−20°C) for 30 min. Haematoxylin and eosin (H&E) staining followed the manufacturer's protocol, with sequential incubation in haematoxylin (7 min), bluing buffer (2 min) and eosin (1 min), with washes in DNase/RNase‐free water between steps. Brightfield images were acquired using a Pannoramic MIDI scanner (3DHISTECH).

#### 4.3.3 Permeabilisation optimisation and library preparation

The Visium Spatial Tissue Optimisation Kit was used to optimise permeabilisation conditions (10× Genomics) by testing multiple permeabilisation durations, followed by fluorescent reverse transcription and tissue removal (56°C, 60 min). The Visium Spatial Gene Expression workflow (10× Genomics) was then used to generate spatial libraries according to vendor instructions. Briefly, released mRNA was captured on the slide by spatially barcoded oligonucleotides before reverse transcription, cDNA amplification and library construction. The final libraries underwent paired‐end 150‐bp sequencing on an Illumina platform.

### 4.4 Immunohistochemistry

IHC staining was applied to formalin‐fixed, paraffin‐embedded tissue sections. After xylene deparaffinisation and graded ethanol rehydration, antigen retrieval was performed in ethylenediaminetetraacetic acid (EDTA) buffer (pH 8.0). After blocking in 5% BSA, sections were incubated overnight at 4°C with primary antibodies targeting GATA3 (mouse, Proteintech, 66400‐1‐Ig, 1:100), CK5 (rabbit, Affinity, AF5479, 1:100), FAP (rabbit, Elabscience, ET1704‐23, 1:1000) and SPP1 (rabbit, Proteintech, 22952‐1‐AP, 1:200). After washing, HRP‐conjugated secondary antibodies were added and 3,3′‐diaminobenzidine (DAB) was used for signal detection. Slides were counterstained with haematoxylin, dehydrated and mounted.

### 4.5 Multiplex immunofluorescence

mIF used sequential tyramide signal amplification (TSA)‐based staining. Two independent four‐colour panels were designed. Panel 1 included CD31 (rabbit, Abcam, ab182981, 1:1000), CD66b (rabbit, Abcam, ab300122, 1:20 000), VEGFA (rabbit, Elabscience, ET1604‐28, 1:3000) and CXCR4 (rabbit, Affinity, AF5279, 1:500). Panel 2 included FAP (rabbit, Elabscience, ET1704‐23, 1:1000), SPP1 (rabbit, Proteintech, 22952‐1‐AP, 1:200), CK5 (rabbit, Affinity, AF5479, 1:100) and FOXP3 (mouse, GeneTex, GT246402, 1:200). For each marker, the primary antibody was added in sequence and left overnight at 4°C; HRP‐conjugated secondary antibody incubation and TSA signal development followed the vendor instructions. Between staining cycles, antibodies were stripped to minimise cross‐reactivity. DAPI was used for nuclear counterstaining, and slides were mounted in antifade medium before imaging.

### 4.6 Cell culture

J82 human UC cells were sourced from the Cell Bank of Type Culture Collection of the Chinese Academy of Sciences; HUVECs were obtained from Procell Life Science & Technology Co., Ltd. Cells were maintained in Dulbecco's modified Eagle's medium (Gibco) containing 10% foetal bovine serum (FBS) and 1% penicillin‒streptomycin under humidified 5% CO_2_ at 37°C. Mycoplasma contamination was routinely excluded before functional assays.

### 4.7 siRNA‐mediated SPP1 knockdown and conditioned medium collection

Igebio synthesised the SPP1‐targeting siRNAs and non‐targeting negative‐control (NC) siRNA. J82 cells were seeded in six‐well plates (2 × 10^5^ cells per well) and transfected once cultures reached 30%‒50% confluence. Transient transfection used Lipofectamine 3000 (Invitrogen). For each well, 50 nM siRNA and Lipofectamine 3000 were diluted in Opti‐MEM Reduced Serum Medium (Gibco, Thermo Fisher Scientific), combined for a 15‐min room‐temperature incubation, and then added to cells in serum‐free medium. After 4‒6 h, fresh complete medium replaced the transfection mixture. For CM collection, complete medium was removed 24 h after transfection, cells were washed with PBS, and serum‐free Opti‐MEM was added for a further 24 h. The resulting CM was collected, centrifuged at 1000 rpm for 5 min to remove debris, and stored at −80°C. Adherent J82 cells from parallel wells were collected for RNA/protein extraction, and knockdown efficiency was evaluated by quantitative real‐time PCR and Western blotting. The siRNA sequences are listed in Table S6.

### 4.8 Quantitative real‐time PCR

Total RNA from transfected J82 cells was purified with the RNAfast200 Total RNA Extraction Kit (Feijie Biotechnology) following the manufacturer's instructions. Genomic DNA was removed before complementary DNA (cDNA) synthesis with the PrimeScript RT reagent Kit with gDNA Eraser (Takara Bio). Quantitative real‐time PCR (qPCR) used the TB Green Premix Ex Taq II (Tli RNaseH Plus) kit (Takara Bio) on a StepOnePlus Real‐Time PCR System (Applied Biosystems). GAPDH served as the endogenous normalisation control, and relative expression was derived with the 2^−ΔΔCt^ method. Primer sequences are listed in Table S7.

### 4.9 Western blotting

Cell lysates were prepared in RIPA buffer (Solarbio) supplemented with 1 mM phenylmethylsulfonyl fluoride (PMSF). Protein was quantified by BCA assay before equal amounts were separated by 10% SDS‒PAGE and transferred to nitrocellulose membranes. Following blocking in Tris‐buffered saline containing Tween 20 (TBST) containing 5% non‐fat milk, membranes were incubated overnight at 4°C with primary antibodies targeting SPP1 (rabbit, Proteintech, 22952‐1‐AP, 1:1000) and β‐actin (mouse, Proteintech, 66009‐1‐Ig, 1:20 000). After washing, membranes received HRP‐conjugated secondary antibodies, and enhanced chemiluminescence was used for detection.

### 4.10 Cell viability assay

Forty‐eight hours after transfection, J82 cells were replated in 96‐well plates at 4000 cells per well. At the indicated time points, 10 µL of Cell Counting Kit‐8 (CCK‐8) reagent (Beyotime) was added per well, followed by a 2‐h incubation at 37°C. Absorbance was measured at 450 nm using a microplate reader, with wells containing medium and reagent only used as blanks.

### 4.11 Transwell migration and invasion assays

Cell motility and invasiveness were tested in 24‐well Transwell chambers equipped with 8.0‐µm pore‐size polycarbonate membrane inserts. For invasion assays, Matrigel was applied to the upper membrane surface (bioGenous) after 1:10 dilution in serum‐free medium and polymerisation at 37°C for 1‒2 h. After 24 h of serum starvation, transfected J82 cells were harvested, washed twice with serum‐free medium, and resuspended in serum‐free Opti‐MEM. For each insert, 6 × 10^4^ cells in 200 µL were added to the upper chamber; uncoated inserts were used for migration and Matrigel‐coated inserts for invasion. The lower chamber contained 600 µL complete medium with 10% FBS. Cultures were incubated for 24 h (migration) or 48 h (invasion); cells remaining on the upper surface were removed, and migrated or invaded cells on the lower membrane surface were fixed with 4% paraformaldehyde, stained with .1% crystal violet, imaged by inverted microscopy, and quantified in ImageJ from at least three random fields per well.

### 4.12 HUVEC tube formation assay

To model angiogenesis‐related crosstalk, Matrigel (50 µL undiluted; bioGenous) was added to each well of a 96‐well plate and allowed to polymerise at 37°C for 30‒45 min. Log‐phase HUVECs were collected, rinsed with serum‐free medium, and resuspended in CM from the indicated J82 groups. HUVECs were plated on polymerised Matrigel at 2.0 × 10^4^ cells per well. After incubation at 37°C for 4‒8 h, capillary‐like networks were imaged by inverted microscopy.

### 4.13 Processing of single‐cell RNA‐seq data

Raw sequencing reads were converted into gene‒barcode matrices with the BD Rhapsody WTA analysis pipeline. Subsequent processing and downstream analysis used Seurat (v5.3.0).^31^ Cells were retained after quality control when they had 300‒6000 detected genes, mitochondrial gene content <30%, and haemoglobin gene content <3%. Expression matrices were log‐normalised with a 10 000 scale factor, and 2000 highly variable genes were retained. Principal component analysis (PCA) was then run, with the first 20 components carried forward. Batch effects were corrected using the Harmony algorithm.^32^ FindNeighbors built the shared nearest‐neighbour graph, and FindClusters performed graph‐based clustering. uniform manifold approximation and projection (UMAP) and t‐SNE were used for two‐dimensional visualisation. Manual cell‐type annotation used canonical lineage markers: B cells (MS4A1, CD79A), plasma cells (IGHG1, IGHG3), cycling cells (MKI67, CCNA2, CCNB2), neutrophils (FCGR3B, CXCR2, CSF3R), myeloid cells (MAFB, CSF1R, CD163, CD14), mast cells (TPSAB1, MS4A2), plasmacytoid DCs (LILRA4, GZMB), mesenchymal cells (COL1A1, COL1A2), ECs (VWF, PECAM1), epithelial cells (EPCAM, KRT19) and T/NK cells (CD3D, CD3E, NKG7). Subclustering analyses were subsequently performed where indicated, and FindAllMarkers was used to define cluster‐specific marker genes. Marker visualisation was performed using ClusterGVis, and gene expression density distributions were displayed using Nebulosa.

### 4.14 Malignant epithelial cell identification

Single‐cell copy number variation (CNV) profiles were inferred with the inferCNV R package, with epithelial cells from adjacent tissues serving as the reference. CNV scores were calculated as the sum of squared gene‐level CNV values from the normalised CNV matrix (scaled to −1 to 1). Pearson correlation coefficients were then computed between each cell's CNV profile and those of high‐CNV cells (top 10% by CNV score). Tumour cells with CNV score >.0005 and correlation coefficient >.2 were classified as malignant and retained for subsequent heterogeneity analyses. To assess the robustness of malignant‐cell identification to reference selection, inferCNV was rerun independently for each tumour sample using immune cells, stromal cells and ECs as putative diploid references. The alternative‐reference and primary analyses showed strong concordance in cell‐level CNV burden, malignant‐cell classification, epithelial subcluster structure, marker profiles and Hallmark pathway activities (Spearman *ρ* = .865; Figure S13).

### 4.15 Assessment of stemness in malignant epithelial cells

CytoTRACE2 was used to estimate stemness in malignant epithelial cells, which integrates gene counts, transcriptional diversity, and a deep learning framework to infer developmental potential.^33^ CytoTRACE2 assigns each cell a score ranging from 0 to 1, where higher values reflect increased stemness and reduced differentiation. Based on these scores, the cluster with the highest stemness signature was selected as the putative developmental origin for subsequent trajectory inference. Analyses were conducted using default parameters.

### 4.16 Trajectory inference

Monocle 2 was used to reconstruct developmental trajectories from single‐cell transcriptomic data.^34^ Highly variable genes (mean expression > .1 detected in at least 10 cells) were selected, and the top 1000 differentially expressed genes (DEGs) ranked by *q*‐value were used for cell ordering. DDRTree was used for dimensionality reduction, and the resulting minimum spanning tree ordered cells along pseudotime. In parallel, Monocle3 was implemented through the SCOP integrated pipeline to assess trajectory robustness.

### 4.17 Gene regulatory network analysis

To characterise transcription factor (TF)‒target regulatory programs in tumour cells, gene regulatory network analysis was performed using pySCENIC.^35^ Raw count matrices were used to infer TF‐centered co‐expression modules with GRNboost, followed by motif enrichment analysis using RcisTarget to define regulons. Regulon activity in individual cells was quantified using AUCell, generating a regulon activity matrix for downstream analysis.

### 4.18 In silico perturbation analysis

scTenifoldKnk was applied to UTUC malignant epithelial Cancer_c0‒Cancer_c4 cells to model SPP1 virtual knockout.^36^ Deionised gene regulatory networks before and after removal of SPP1 outgoing edges were compared by manifold alignment, and the resulting differentially regulated genes were subjected to Gene Ontology (GO) and Kyoto Encyclopedia of Genes and Genomes (KEGG) enrichment analyses.

### 4.19 Computational analysis of UTUC ST data

Space Ranger (10× Genomics) processed raw ST data and aligned reads to the GRCh38 human reference genome. Default parameters generated gene‒barcode matrices and spatial coordinates. Cell‐type composition was inferred using cell2location^37^ with pre‐annotated scRNA‐seq data as reference; the model was trained for 50 000 epochs to estimate cell‐type‐specific abundances across spatial spots. Spots were filtered based on posterior abundance estimates (q05). For each section, cell‐type abundances were summed across retained spots and normalised to the total inferred cellular abundance for downstream analyses.

Spatial niches were defined by Leiden clustering of a *k*‐nearest neighbour (kNN) graph constructed from cell2location‐inferred spot‐level cell‐type abundance profiles. Spatial co‐localisation between cell subtypes was quantified using cell2location‐derived proportions: proportions were discretised into three ordinal levels, and a co‐localisation score was defined as the product of the ranked abundances. Spatial ligand‒receptor co‐localisation was visualised using SpaGene, which computes a kNN‐based neighbourhood interaction score for each spot.

The SPP1^+^ TAM‒FAP^+^ myCAF and VEGFA^+^ TAN‒CXCR4^+^ tip EC programs were quantified in UTUC sections using multiscale pair burden, within‐section permutation‐based observed‐to‐expected enrichment, and signed‐distance profiles along the inferred tumour‒boundary axis. Within‐spot, immediate‐neighbourhood, and extended‐neighbourhood scales corresponded to a 55‐µm capture diameter and center distances of 100 µm and 173‒200 µm, respectively; pair burden was the mean symmetric geometric abundance of the two component states across eligible spot pairs. Within each section, O/E enrichment was defined as the observed pair burden divided by the mean pair burden from 500 positional permutations of one component. Tumour‐reference spots were defined by a section‐specific Otsu threshold of the summed malignant epithelial fraction, after which stGrads inferred the tumour interface and signed distance; negative and positive distances denoted the tumour and non‐tumour sides, respectively. All comparisons used the section as the analytical unit. For the SPP1^+^ TAM‒FAP^+^ myCAF program, effector immune scores were additionally summarised across the outer non‐tumour, boundary and adjacent tumour compartments.

### 4.20 Tumour microenvironment crosstalk analysis

CellChat (v1.6.1 for scRNA‐seq and v2.1.2 for spatial analysis) with CellChatDB.human was used to infer candidate ligand‒receptor interactions among annotated cell states.^38^ Overexpressed signalling genes were identified using the default thresholds (thresh.pc = 0, thresh.fc = 0 and thresh.p = .05), followed by identification of overexpressed ligand‒receptor pairs. For scRNA‐seq data, communication probabilities were estimated from normalised expression using the default trimean and 100 permutations; interactions involving groups with fewer than 10 cells were excluded. For spatial data, communication probabilities were calculated from normalised SCT expression using a 10% truncated mean and 100 permutations, with distance weighting enabled (interaction range = 250 µm; scale.distance = 3.65). Contact‐dependent signalling was restricted to a 100‐µm range, and interactions involving groups with fewer than 10 spots were excluded. Interactions with permutation *p* < .05 were retained and aggregated at the signalling pathway level.

NicheNet was used to prioritise ligands from SPP1^+^ TAMs to FAP^+^ myCAFs.^39^ The receiver gene set comprised genes consistently upregulated in MI versus NMI tumours, MI tumours versus adjacent tissues, and FAP^+^ myCAFs versus other CAF states. Ligands were ranked according to their predicted ability to regulate this receiver gene program, and the corresponding ligand‒target relationships were extracted from the NicheNet prior model.

### 4.21 Bulk transcriptomic deconvolution

To enable cross‐modal integration, scRNA‐seq data were used as a reference for bulk transcriptomic deconvolution. The raw count matrix and manually annotated cell‐type labels derived from scRNA‐seq were exported to CIBERSORTx (https://cibersortx.stanford.edu/) to construct a signature matrix. Relative cell‐type abundances in each bulk RNA‐seq sample were subsequently estimated using default parameters. The inferred cell fractions were used for downstream phenotype association and survival analyses.

### 4.22 Cell‐type‐specific genetic susceptibility analysis

To map inherited susceptibility signals to specific cellular compartments in UTUC, we integrated UTUC GWAS summary statistics with scRNA‐seq data using scPagwas.^40^ Gene‐level association signals were aligned with cell‐type‐specific transcriptional profiles to quantify genetic risk enrichment across annotated cellular subsets. Statistical significance was determined by permutation testing with Benjamini‒Hochberg correction, and false discovery rate (FDR)‐adjusted *p*‐values were reported.

### 4.23 Phenome‐wide Mendelian randomisation analysis

PheW‐MR was used to assess the druggability and pleiotropic effects of SPP1 across human traits. Genetic instruments were derived from expression quantitative trait locus (eQTL) data, and outcome summary statistics were obtained from UK Biobank GWAS (*N* = 456 348). After quality control (minimum case number > 500), 679 of 1403 binary phenotypes were included. Causal associations were estimated using the summary‐data‐based Mendelian randomisation (SMR) method, with HEIDI *p* > .1 applied to exclude linkage. Statistical significance was defined as FDR‐adjusted *p* < .05. Phenotypes were categorised according to the PheCode system (Table S8).

### 4.24 Functional enrichment and pathway activity analysis

TME immune and stromal signatures were calculated using ESTIMATE. For each bulk sample, gene set variation analysis (GSVA) calculated basal/luminal scores from the BASE47 gene set reported by Damrauer et al.^12^ Seurat FindMarkers was used to identify DEGs between single‐cell subpopulations. Functional differences across cell subsets were evaluated with clusterProfiler^41^ using gene set enrichment analysis (GSEA) together with GO and KEGG enrichment analyses. The ratio of observed to expected cell numbers (*R*_o/e_) was calculated using a negative binomial model to quantify inter‐group compositional differences among cell types; *R*_o/e_ > 1 indicated enrichment relative to expectation, whereas *R*_o/e_ < 1 indicated relative depletion. For single‐cell data, AUCell estimated gene set activity from the recovery area of ranked expression profiles. Pathway activities across cell subtypes were further evaluated using PROGENy, a perturbation‐based pathway inference method covering 12 canonical cancer‐related signalling pathways. For ST data, gene set activity was assessed using SpaCET to obtain spot‐level enrichment scores.

### 4.25 External validation in public BCa datasets

For TCGA‐BLCA and GSE32894, the BASE47‐derived basal‒luminal score was defined as the GSVA basal score minus the luminal score. ESTIMATE scores were compared between basal‐high and luminal‐high tumours, and linear models evaluated the effects of subtype score, stage and their interaction. Public BCa scRNA‐seq datasets were processed with Seurat and annotated using canonical lineage markers. After filtering and normalisation of GSE299573 Visium HD 8‐µm bins, RCTD was used to infer major cell‐type proportions, and module scores were calculated for luminal, basal, SPP1^+^ TAM, FAP^+^ myCAF, T‐cell‐inflamed/cytotoxic, immune‐suppressive, and myeloid‐suppressive programs. In GSE296955 GeoMx WTA data, the composite SPP1^+^ TAM‒FAP^+^ myCAF niche score was defined as the sum of the *z*‐scored component signatures, and associations were assessed by Spearman correlation at the ROI and aggregated levels. In GSE319536, the joint SPP1^+^ TAM‒FAP^+^ myCAF burden derived from RCTD‐inferred cell‐state proportions was modelled along the within‐section epithelial basal‒luminal axis using section‐specific generalised additive models and summarised across 22 Visium sections.

### 4.26 Survival and prognostic modelling

DSS was used as the primary endpoint. The log‐rank test compared Kaplan‒Meier survival curves. Univariable and multivariable Cox proportional hazards models estimated HRs and 95% CIs. Five‐year time‐dependent receiver operating characteristic (ROC) analysis was performed using the timeROC package to evaluate prognostic performance. ROC curves for subtype classification were generated using the pROC package. Expression thresholds were selected with maximally selected rank statistics in the survminer package.

### 4.27 Statistical analysis

Bioinformatic and statistical analyses used R (v4.3.1) and Python (v3.10.11). Where applicable, the Benjamini‒Hochberg FDR method adjusted for multiple testing. Associations between continuous variables were evaluated with Pearson correlation. All tests were two‐sided, and *p*‐values below .05 denoted statistical significance. Wet‐lab experiments were performed with independent biological replicates, and group comparisons used two‐sided Student's *t*‐tests or one‐way analysis of variance, as appropriate. Associations with muscle invasion in the Japan‐UTUC cohort were evaluated using logistic regression with adjustment for age and sex.

## AUTHOR CONTRIBUTIONS

Qi Zhang, Zhipeng Wen, Ziyang Yang and Xuezhi Long contributed equally to this work. Qi Zhang, Ziyang Yang, Zhipeng Wen and Yufei Liu conceptualised the study. Qi Zhang, Ziyang Yang and Xuezhi Long developed the methodology. Zhipeng Wen, Yueting Huang, Jiahao Cheng and Yubo Wang acquired the data. Zhipeng Wen performed the SPP1 knockdown and functional validation experiments. Qi Zhang, Xuezhi Long, and Jieshen Xian analysed and interpreted the data. Qi Zhang, Ziyang Yang, Xuekun Zhang and Gengjia Chen wrote the original draft. Xuezhi Long, Xuekun Zhang, Yufei Liu, Tuxiong Huang and Di Gu reviewed and edited the manuscript. Tuxiong Huang and Di Gu supervised the project. All authors reviewed and approved the final manuscript.

## CONFLICT OF INTEREST STATEMENT

The authors declare they have no conflicts of interest.

## ETHICS STATEMENT

This study was approved by the Ethics Committee of the First Affiliated Hospital of Guangzhou Medical University (approval no. ES‐2024‐171‐01) and was conducted in accordance with the Declaration of Helsinki. Informed consent was obtained from all participants involved in the study.

## Supporting information



Supporting Information

Supporting Information

Supporting Information

Supporting Information

Supporting Information

Supporting Information

Supporting Information

Supporting Information

Supporting Information

## Data Availability

The datasets generated and analysed in this study are available from the corresponding author upon reasonable request. Public datasets and accession numbers used in this study are listed in Table S5. Analytical code is publicly available at https://github.com/zzzqi123/Basal‐UTUC‐spatial‐ecosystems.
