# Supplementary material for: SPP1‐driven spatial niche remodelling defines aggressive progression in the basal subtype of upper tract urothelial carcinoma
Source: Clin Transl Med. 2026 Aug 24;16(8):e70782. doi: 10.1002/ctm2.70782 (PMC13501109; doi:10.1002/ctm2.70782)
Supplement: Supplementary file 1 [file CTM2-16-e70782-s008.docx]

**Supplementary Figures**

**Supplementary Figure Overview**

**S1.** Molecular subtype distribution and tumor microenvironment features across stages

**S2.** Single-cell RNA sequencing quality control, marker validation, and cell-type composition

**S3.** UTUC GWAS risk mapping across single-cell populations

**S4.** Spatial transcriptomic profiling of Basal UTUC across pathological stages

**S5.** Malignant epithelial states, developmental trajectories, spatial localization, and clinical outcome

**S6.** Malignant-cell transcriptional programs, spatial organization, external validation, and clinical association

**S7.** Myeloid subclusters, pseudotime progression, spatial co-localization, and survival

**S8.** Lymphoid composition, functional programs, spatial organization, and survival

**S9.** Detailed CD4^+^ and CD8^+^ T-cell states and lymphoid clinical associations

**S10.** Mesenchymal and endothelial heterogeneity, spatial programs, and survival

**S11.** Bulk bladder cancer validation of the SPP1-associated Basal immune–stromal program

**S12.** Single-cell bladder cancer validation of SPP1^+^ TAM, FAP^+^ myCAF, and CXCR4^+^ tip-like endothelial compartments

**S13.** inferCNV reference-sensitivity and malignant-subcluster concordance analyses

**S14.** Tumor-boundary inference and spatial-niche functions

**Supplementary Figure S1**

**Supplementary Figure S1. Molecular subtype distribution and tumor microenvironment–related features across stages.**

(A) Distribution of molecular subtypes across pathological stages (non–muscle-invasive, NMI; muscle-invasive, MI). (B) Tumor purity compared between Luminal and Basal tumors within each stage (NMI and MI). (C) Expression levels of representative immune activation markers (CD3E, CD4, IL7R, CD8A, GZMK), immune checkpoint and exhaustion markers (LAG3, PDCD1, FOXP3, CTLA4), myeloid-associated markers (SPP1, CSF1R), and stromal markers (COL1A1, COL6A1, VIM, FAP, PDGFRA) compared between Luminal and Basal tumors within each stage. (D) Sankey plot showing the relative proportions of major cell types across adjacent, NMI, and MI samples.

**Supplementary Figure S2**

**Supplementary Figure S2. Quality control, marker validation, and cell-type distribution of single-cell RNA sequencing data.**

(A) Quality control metrics of single-cell RNA sequencing across samples, including the number of detected genes (nFeature RNA), total UMI counts (nCount RNA), percentage of mitochondrial transcripts (MT percent), and percentage of hemoglobin transcripts (HB percent). (B) UMAP density feature plots showing the expression of representative lineage markers used for cell-type annotation. (C) Proportions of major cell types across adjacent, non–muscle-invasive (NMI), and muscle-invasive (MI) samples.

**Supplementary Figure S3**

**Supplementary Figure S3. Integration of UTUC GWAS signals with single-cell transcriptomic profiles using scPagwas.**

(A) t-SNE projection of annotated cell types from single-cell RNA sequencing data. (B) GWAS risk-associated cell mapping inferred by scPagwas, with color intensity representing –log_10_(FDR). (C) Summary of cell-type-specific genetic risk associations with corresponding FDR values.

**Supplementary Figure S4**

**Supplementary Figure S4. Spatial transcriptomic analysis of Basal UTUC across pathological stages.**

(A) Representative hematoxylin and eosin (H&E) images of non–muscle-invasive (NMI) and muscle-invasive (MI) Basal tumors subjected to spatial transcriptomic profiling. (B) Spatial niche clustering showing the distribution of identified niches within each sample. (C) Spatial maps of deconvoluted cell types inferred using matched single-cell reference data. (D) Spatial visualization of the Basal gene signature score.

**Supplementary Figure S5**

**Supplementary Figure S5. Developmental trajectories define functionally and spatially distinct malignant epithelial states in UTUC.**

(A) InferCNV heatmap showing large-scale chromosomal copy number variation patterns used to distinguish malignant epithelial cells from non-malignant cells. (B) UMAP visualization of five malignant epithelial subclusters (Cancer_c0–Cancer_c4). (C) Differentiation potential inferred by CytoTRACE2 (left) and pseudotime trajectory reconstructed by Monocle3 (right), illustrating developmental progression among malignant epithelial cells. (D) Density plots showing expression distribution of representative basal-associated markers (KRT6A and KRT5) across malignant epithelial cells. (E) Spatial transcriptomic mapping of malignant epithelial subclusters across tumor sections. (F) Quantification of spatial niche distribution demonstrating distinct localization patterns of Cancer_c0 and Cancer_c3. (G) Kaplan–Meier analysis of disease-specific survival stratified by Cancer_c0 and Cancer_c3 signature expression. (H) Transcription factor regulon analysis showing the top 6 transcription factors enriched in Cancer_c0 and Cancer_c3 subclusters. (I) Gene set enrichment analysis of Cancer_c0-associated gene programs. (J) Heatmap showing pathway activity scores inferred by PROGENy across malignant epithelial subclusters.

**Supplementary Figure S6**

**Supplementary Figure S6. Developmental trajectories, transcriptional programs, spatial organization, and clinical association of malignant epithelial subclusters.**

(A) Heatmap showing the top 10 marker genes for each malignant epithelial subcluster (Cancer_c0–c4), with representative enriched Gene Ontology biological process terms shown alongside each cluster. (B) PCA-based trajectory inference illustrating two differentiation lineages among malignant epithelial cells. (C) Monocle3 trajectories projected onto the UMAP and colored by malignant epithelial subcluster (top) or trajectory state (bottom). (D) Density maps showing the regulon activity of representative transcription factors enriched in Cancer_c0 (TP53 and RXRA) and Cancer_c3 (CEBPG and MXI1). (E) Gene set enrichment analysis of Cancer_c3-upregulated genes, highlighting TGF-β signaling, KRAS signaling, hypoxia, and glycolysis. (F) Section-specific generalized additive model trajectories of standardized Cancer_c3 abundance along signed distance from the inferred tumor boundary in the NMI-Basal, MI-Basal-1, and MI-Basal-2 sections. Negative and positive distances indicate the tumor and non-tumor sides, respectively, and the dashed vertical line marks the inferred tumor boundary. (G) Spatial trajectories of Cancer_c0, Cancer_c3, and the grouped Cancer_c1/c2/c4 programs along the within-section epithelial basal–luminal continuum in 22 bladder cancer Visium sections (GSE319536). Lines show equal-section smoothed estimates with 95% confidence intervals; paired section-level comparisons of the basal-minus-luminal shifts are indicated. (H) Odds ratios for muscle invasion per 1-SD increase in the Cancer_c3 score in the Japan-UTUC cohort, estimated using univariable and age- and sex-adjusted logistic regression models.

**Supplementary Figure S7**

**Supplementary Figure S7. Transcriptional and spatial characterization of myeloid subclusters in UTUC.**

(A) Dot plot showing canonical marker gene expression across myeloid subclusters. Dot size represents the percentage of expressing cells and color intensity indicates scaled expression level. (B) Heatmap showing the top 10 differentially expressed genes for each myeloid subcluster, with corresponding Gene Ontology biological process (BP) enrichment analysis. (C) Density plots illustrating changes in myeloid subcluster abundance along pseudotime progression. (D) Spatial transcriptomic mapping showing co-localization of Mono_c3_CCR2 monocytes and Macro_c0_SPP1 macrophages, alongside TAM M2 signature score distribution. (E) Kaplan–Meier survival analysis of disease-specific survival stratified by representative dendritic cell subclusters.

**Supplementary Figure S8**

**Supplementary Figure S8. Transcriptional heterogeneity and spatial organization of lymphoid subclusters in UTUC.**

(A) UMAP visualization of major lymphoid populations, including B cells, CD4^+^ T cells, CD8^+^ T cells, NK cells, and plasma cells. (B) Functional gene set enrichment of CD4^+^ T cell subclusters categorized into apoptosis, differentiation, function, and metabolism modules. (C) Kaplan–Meier analysis of disease-specific survival stratified by representative lymphoid subcluster signatures. (D) Relative overrepresentation (Ro/e) analysis of CD8^+^ T cell subclusters across adjacent tissue, NMI, and MI groups. (E) Spatially resolved enrichment scores of T cell activation, T cell inhibition, Treg, and TLS gene sets in representative NMI and MI tumors.

**Supplementary Figure S9**

**Supplementary Figure S9. Transcriptional and functional heterogeneity of lymphoid subclusters in UTUC.**

(A) UMAP visualization of CD4^+^ T cell subclusters across samples. (B) Heatmap showing the top 10 differentially expressed genes for each CD4^+^ T cell subcluster with corresponding functional annotation. (C) Relative overrepresentation (Ro/e) analysis of CD4^+^ T cell subclusters across adjacent tissue, NMI, and MI groups. (D) UMAP visualization of CD8^+^ T cell subclusters. (E) Heatmap showing the top 10 differentially expressed genes for each CD8^+^ T cell subcluster with associated biological process annotation. (F) Functional gene set enrichment analysis of CD8^+^ T cell subclusters, including pathways related to activation, exhaustion, cytotoxicity, metabolism, and stress response. (G) Kaplan–Meier analysis of disease-specific survival stratified by representative B cell subclusters. (H) Correlation analyses between lymphoid subcluster signatures and representative immune or basal-associated markers.

**Supplementary Figure S10**

**Supplementary Figure S10. Transcriptional and spatial heterogeneity of mesenchymal and endothelial compartments in UTUC.**

(A) Heatmap showing the top 10 marker genes of mesenchymal subclusters with corresponding biological process (BP) enrichment analysis. (B) Spatial enrichment score of the myogenesis gene signature in MI-Basal tumors. (C) Hallmark gene set enrichment analysis of the CAF_c3_FAP subcluster. (D) UMAP visualization illustrating endothelial heterogeneity across distinct vascular states. (E) Dot plot showing expression of canonical endothelial markers across endothelial subclusters. (F) Hallmark gene set enrichment analysis of the Endo_c1_CXCR4 tip-like endothelial subcluster. (G) Kaplan–Meier analysis of disease-specific survival according to mesenchymal and endothelial subcluster–associated signatures.

**Supplementary Figure S11**

**Supplementary Figure S11. Bulk transcriptomic validation of the SPP1-associated Basal immune–stromal program in bladder urothelial carcinoma.**

(A) Stromal and immune scores compared between Luminal-high and Basal-high tumors within NMI and MI groups. (B) FAP and SPP1 expression compared between Luminal-high and Basal-high tumors within NMI and MI groups. (C) Correlations between SPP1 expression and Basal subtype score, immune score, and stromal score. (D) Receiver operating characteristic (ROC) curves evaluating Luminal–Basal subtype classification using SPP1 and canonical subtype markers GATA3 and KRT5. (E) Kaplan–Meier survival analyses of disease-specific survival (DSS) in GSE32894 and overall survival (OS) in TCGA-BLCA according to SPP1 expression. Statistical values are shown in the corresponding panels.

**Supplementary Figure S12**

**Supplementary Figure S12. Single-cell validation of bladder urothelial carcinoma cell-type annotations and SPP1/FAP/CXCR4-associated compartments (GSE222315 and GSE267718).**

(A) UMAP visualization of major cell lineages in a public bladder urothelial carcinoma single-cell dataset. (B) Dot plot showing canonical lineage marker expression across annotated cell types. (C) Dot plot of neutrophil-associated markers across annotated cell types; these markers did not resolve a discrete neutrophil population in this dataset. (D) Reclustering of myeloid cells, SPP1 density mapping, and marker expression distinguishing SPP1^+^ TAM from other SPP1^-^ myeloid cells. (E) Reclustering of fibroblasts, FAP density mapping, and marker expression distinguishing FAP^+^ myCAF from other FAP^-^ fibroblasts. (F) Reclustering of endothelial cells, CXCR4 density mapping, and marker expression distinguishing tip-like endothelial cells from other endothelial cells.

**Supplementary Figure S13**

**Supplementary Figure S13. Robustness of malignant epithelial subcluster assignments and functional programs under alternative inferCNV reference selection.**

(A) Original inferCNV analysis using adjacent epithelial cells as the reference. (B) Revised inferCNV analysis using within-sample immune, stromal, and endothelial cells as non-epithelial references. (C) Concordance of cell-level CNV burden percentile ranks between the original and revised analyses. The solid line denotes the linear fit and the dashed line denotes identity. (D) Patient-specific Spearman correlations of cell-level CNV burden ranks between the two analyses. (E) UMAP visualization of malignant epithelial subcluster assignments derived from the original and revised inferCNV results. (F) UpSet plot showing the overlap of malignant epithelial cells identified by the original and revised inferCNV analyses. (G) Spearman correlation matrix of subcluster marker expression profiles; outlined cells indicate corresponding subcluster pairs. (H) Concordance of Hallmark normalized enrichment scores for Cancer_c0 and Cancer_c3 between the original and revised analyses. Representative pathways are labeled, and statistical values are shown in the corresponding panels.

**Supplementary Figure S14**

**Supplementary Figure S14. Tumor-boundary inference and functional characterization of spatial niches in Basal UTUC.**

(A) Spatial maps of signed distance from the inferred tumor boundary in the NMI-Basal, MI-Basal-1, and MI-Basal-2 sections. Section-specific Otsu thresholds of the broad malignant-cell fraction were used to define tumor reference spots, after which the tumor interface and signed distance were inferred using stGrads. (B) Hallmark gene set enrichment analysis based on an integrated ranked gene list for each spatial niche. Representative positively enriched programs are shown, with normalized enrichment scores and nominal and adjusted P values indicated. (C) Adjusted spatial associations between niche burden and pathway scores in the two MI-Basal sections. Models included both niche burdens and were adjusted for library size, total inferred cell abundance, and malignant-cell fraction. Diamonds and horizontal bars indicate pooled standardized partial β estimates and 95% confidence intervals, respectively; q values are shown.
